# Glycerol Triacetate-Based Flame Retardant High-Temperature Electrolyte for the Lithium-Ion Battery

**DOI:** 10.1021/acsami.4c02323

**Published:** 2024-05-06

**Authors:** Xinsheng Wu, Tong Liu, Young-Geun Lee, Jay. F. Whitacre

**Affiliations:** † Department of Materials Science and Engineering, Carnegie Mellon University, 5000 Forbes Avenue, Pittsburgh, Pennsylvania 15213, United States; ‡ Department of Chemistry, Carnegie Mellon University, 4400 Fifth Avenue, Pittsburgh, Pennsylvania 15213, United States; § Scott Institute for Energy Innovation, Carnegie Mellon University, 5000 Forbes Avenue, Pittsburgh, Pennsylvania 15213, United States

**Keywords:** high-temperature electrolyte, battery, Li-ion battery, electrolyte, safety

## Abstract

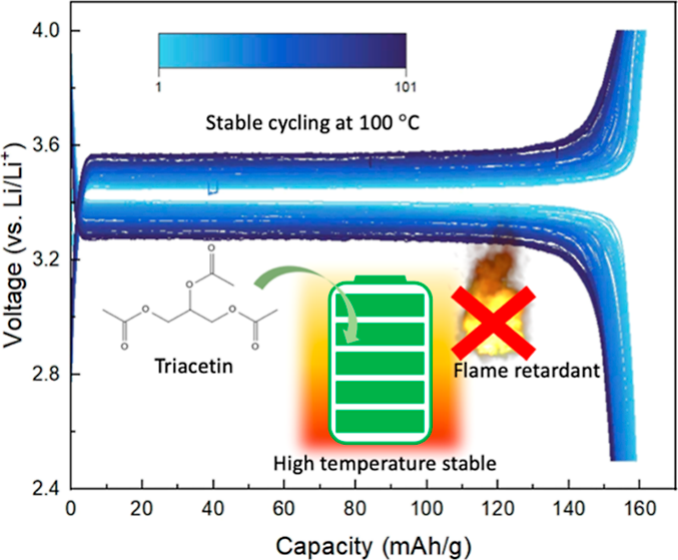

Rechargeable batteries that can operate at elevated temperatures (>70 °C) with high energy density are long-awaited for industrial applications including mining, grid stabilization, naval, aerospace, and medical devices. However, the safety, cycle life, energy density, and cost of the available high-temperature battery technologies remain an obstacle primarily owing to the limited electrolyte options available. We introduce a flame-retardant electrolyte that can enable stable battery cycling at 100 °C by incorporating triacetin into the electrolyte system. Triacetin has excellent chemical stability with lithium metal, and conventional cathode materials can effectively reduce parasitic reactions and promises a good battery performance at elevated temperatures. Our findings reveal that Li–metal half-cells can be made that have high energy density, high Coulombic efficiency, and good cycle life with triacetin-based electrolytes and three different cathode chemistries. Moreover, the nail penetration test in a commercial-scale pouch battery using this new electrolyte demonstrated suppressed heat generation when the cell was damaged and excellent safety when using the triacetin-based electrolyte.

## Introduction

Due to the substantial decrease in the cost of lithium-ion batteries (LIBs), the number of electric vehicles has experienced significant expansion in recent years. Presently, over 80% of the LIBs produced are intended for transportation.^1^ At the same time, consumers are demanding battery packs with higher energy density and lower danger levels to reduce the range anxiety and safety concerns.^2^ Currently, most commercial LIBs use organic electrolytes with a low boiling point (<140 °C) and a low flash point (<40 °C),^3^ making the battery not only perform worse at elevated temperatures but also pose a high risk of thermal runaway under abuse conditions.^4^ High-energy rechargeable batteries also have a high demand in many industrial applications that require continuous operation in extreme environments, like the mining industry.^5,6^ To address these issues, many efforts have been devoted to finding new electrolyte systems that are nonflammable^7−9^ or self-extinguishing^10^ electrolytes, which can allow the battery to operate in more extreme conditions. However, there is still a lack of understanding about the battery performance at extreme temperatures above 80 °C.^8^ More importantly, many papers have focused on using concentrated electrolytes^11,12^ or highly fluorinated electrolytes,^13,14^ which have a high cost for commercialization and are also difficult to fabricate on a large scale. Additionally, the solid-state electrolyte with a nonflammability nature is considered the ultimate goal for the lithium-ion battery industry, but at their cost, ionic conductivity and compatibility with electrodes remain huge problems.^15^ Alternative electrolytes with low cost, high stability, and high performance are needed to meet the demand of high-temperature batteries.

Batteries operating at higher temperatures typically experience much faster capacity decay than that at lower temperatures, and three main factors are acknowledged to contribute to the decay: electrolyte evaporation, solid electrolyte interface (SEI), and cathode electrolyte interface (CEI) layer decomposition, and cathode failure. At higher temperatures, conventional low boiling point electrolytes evaporate and decompose, creating a locally dry electrode area with a high resistance. This dry electrode area leads to uneven current distribution and further uneven lithium plating.^16^ The surface layer at the anode and the cathode will also decompose at elevated temperatures, especially the organic components.^17−19^ The exposure of the fresh cathode/anode surface will drive the further reaction of the electrolyte with the electrode and create a thicker SEI/CEI layer, exaggerating the consumption of the electrolyte.^20,21^ Moreover, cathode active materials will also play an important role in capacity decay at higher temperatures. As temperatures increase, most cathode materials become less stable as lithium leaves their structure, exacerbating the transition-metal ion dissolution problem.

The transition-metal dissolution problem not only leads to the pulverization of the active material but also catalyzes the electrolyte decomposition, eventually leading to complete battery failure at higher temperatures.^22,23^ Additionally, self-discharging problems will become more severe at higher temperatures since the mobility of the lithium-ion will also be higher and will also contribute to capacity decay.^24^ To improve the high-temperature performance of the battery, it is necessary to have a better electrolyte system with a higher boiling point and higher stability that can help us understand the battery failure mechanism at higher temperatures and enable a safer battery for application.^3,25,26^

The long-term storage of batteries at elevated temperatures is another challenge for high-temperature batteries that have been studied extensively. Batteries stored at higher temperatures experience spontaneous electron diffusion from the cathode to the electrolyte, which triggers electrolyte decomposition and cathode degradation.^27^ The decomposition and reconstruction of the SEI/CEI layer also consume active lithium in the cell and cause irreversible capacity decay at higher temperatures during long-term storage.^28^ For these reasons, long-term storage properties also need to be considered in the design of new high-temperature electrolytes.

Here, we introduce glycerol triacetate (GTA, also called triacetin) as a new flame-retardant high-temperature electrolyte solvent to replace conventional carbonate electrolytes in higher-temperature operating environments. GTA has a high boiling point (258 °C) and high flash point (138 °C), along with high stability with the lithium metal,^29^ making it an ideal candidate for batteries requiring severe thermal conditions. Moreover, GTA is widely used in the biomedical and food industries due to its low toxicity, ecofriendliness, and low cost; it can be easily sourced and readily implemented in commercial battery systems. In this work, we combined GTA with ethylene carbonate (EC) and fluoroethylene carbonate (FEC) as cosolvents with lithium bis(trifluoromethanesulfonyl)imide (LiTFSI) and lithium difluoro(oxalate)borate (LiDFOB) as lithium salts to evaluate the possibility of using GTA as a high-temperature electrolyte solvent. FEC and LiDFOB were reported to be beneficial to the battery performance as the filming agent on both cathode and anode sides.^30−32^ The adoption of FEC and LiDFOB could help build a robust SEI/CEI layer, preventing dendrite formation on the anode side and cathode failure on the cathode side at elevated temperatures. Based on these considerations, two combinations G1 [1 M LiTFSI in GTA/EC = 1:1 (v/v)] and G2 [0.5 M LiDFOB in GTA/FEC = 5:2 (v/v)] are mainly tested in our work and compared with commercial electrolyte COM [1 M LiPF6 in EC/DEC/DMC = 1:1:1 (v/v/v)]. The performances of the battery with three different cathode chemistries, nickel manganese cobalt (NCM), lithium iron phosphate (LFP), and fluorinated carbon (CF*x*)—were demonstrated at 100 °C. The flame-retardant ability of this electrolyte system was also demonstrated in a commercial-level pouch cell. The results showed improved battery performance at extreme temperatures (100 °C) with enhanced safety.

## Experimental Section

### Materials and the Preparation of the Electrolytes

PVDF, NCM523, and LFP powders were purchased from MTI Co., Ltd. in this work. LiDFOB (99%) and LiTFSI (99.99%) lithium salts were purchased from Sigma-Aldrich. FEC (98%) and GTA (99%) were purchased from Alfa Aesar, and EC was purchased from Gelon Co., Ltd. All of the reagents were used as received. The electrolyte solvents were first mixed at the corresponding volume ratio, and the lithium salt was then added into the solvent and stirred overnight at 60 °C in the argon-filled glovebox to obtain a homogeneous electrolyte mixture.

### Fabrication of Cathode Electrodes

The cathode electrodes were prepared through a doctor blade method as follows. The PVDF powder was first dissolved in NMP to be a 4 wt % solution. Cathode active materials (including LFP, NCM523, and CF*x*), Super P carbon black, and the PVDF solution were then mixed using the mortar and pestle with a weight ratio of 8:1:1 for 10 min to homogenize. The as-prepared slurry was then doctor-bladed onto aluminum foil and dried overnight at 100 °C in a vacuum oven. The dry electrode has a cathode active material loading of ∼5 mg/cm^2^ and was punched out to a 12 mm diameter disk for battery assembly.

### Battery Assembly and Electrochemical Tests

CR2032 coin cells were used and assembled in an argon-filled glovebox for both half cells and symmetric cells. All cells used 100 μL of the electrolyte and lithium foils with a thickness of 600 μm and a Celgard 3401 surfactant-coated separator. The NCM523||graphite pouch cells were also assembled in an argon-filled glovebox. Pouch cells were first dried at 100 °C overnight and then transferred into the argon-filled glovebox and injected 1000 μL of the electrolyte for each pouch cell. Cyclic voltammetry (CV) and EIS tests were conducted using a three-electrode beaker cell system with two platinum electrodes as the working and counter electrodes, and one lithium metal electrode was used as the reference electrode. CV scans for each electrolyte were conducted with a scan rate of 0.01 mV/s at 25 °C and a voltage window between 4.5 and 0 V vs Li/Li^+^. The frequency range of the EIS tests was from 1 MHz to 100 mHz with a sinus amplitude of 100 mV. Both EIS and CV were conducted on a Biologic. Long-term galvanostatic cycling tests for both coin cells and pouch cells were conducted using test instruments from LAND electronic Co., LTD in a high-temperature oven at their corresponding temperatures. The voltage windows of the galvanostatic cycling tests are 3.0–4.3 V vs Li/Li^+^ for NCM523 cells and 2.5–4.0 V vs Li/Li^+^ for LFP cells.

### Flammability Tests

The flammability tests of the full pouch cells were conducted at a room temperature of 23 °C. Pouch cells using G2 and COM electrolyte for the flammability are both cycled for three cycles and end with a fully charged state. The nail penetration test was conducted by using a needle with a diameter of 0.5 mm. The infrared images and video were captured by using a FLIR One PRO thermal camera.

### Material Characterization

Differential scanning calorimetry (DSC) was measured with a TA Instrument Q-20 under nitrogen with a heating rate of 2.5 °C/min, and the mass of the sample was 10 mg for each test. TGA was measured with a TA Instrument Q-50 with a heating rate of 5 °C/min under nitrogen. SEMs were conducted using a Tescan Mira3 instrument at an accelerating voltage of 15 kV with a spot size of 8 nm and beam intensity of 13. The magnification of the photos ranged from 15,000 to 25,000×. ^1^H NMR spectroscopy measurements were performed on a Bruker Advance 300 MHz spectrometer using CDCl_3_ as the solvent. Transmission electron microscopy (TEM) image was collected using Tecnai F20.

## Results and Discussion

### Physical Properties of the GTA-Based Electrolytes

**Figure 1:**
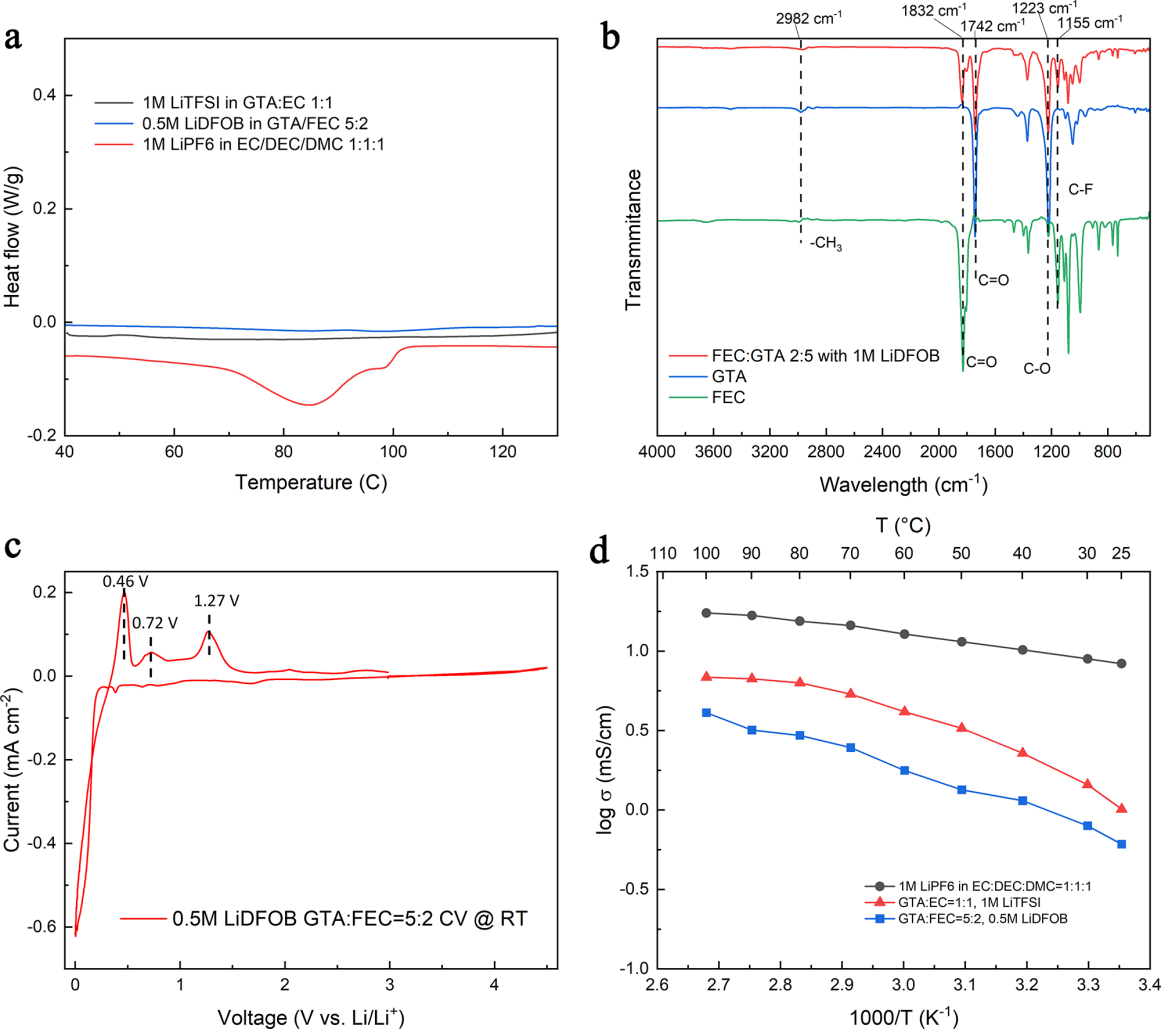
Physical properties of the GTA-based electrolyte. (a) DSC results of G1, G2, and COM electrolyte. (b) FTIR spectrum of the G2 electrolyte and FEC, GTA. (c) CV scan of the G2 electrolyte in a three-electrode cell. (d) Arrhenius plot of the ionic conductivity of G1, G2, and COM electrolytes.

To assess the stability of the electrolyte at high temperatures, DSC was performed on pure G1, G2, and COM electrolytes. Figure 1a demonstrates that the G1 and G2 electrolytes did not exhibit any exothermic or endothermic reaction peak before 130 °C. However, the COM electrolyte showed a strong endothermic peak at 80 °C, which can be attributed to the evaporation of the electrolyte. Thermogravimetric analysis (TGA) was also performed to investigate the evaporation behavior of these electrolyte systems. As demonstrated in Supporting Information Figure S1, both G1 and G2 show low vapor pressure with negligible evaporation below 100 °C. In contrast, the commercial electrolyte begins to evaporate significantly even at room temperature, indicating a high vapor pressure. These results validate that GTA provides the electrolyte with a high boiling point and good stability.

Figure 1b shows the FTIR spectra of the G2 electrolyte, pure GTA, and pure FEC. The peaks at 1155 and 1742 cm^–1^ are attributed to the FEC, corresponding to C–F and C=O stretching, respectively. The peaks at 1223 and 1832 cm^–1^ are attributed to the FEC, corresponding to C–O and C=O stretching, respectively. After mixing and adding the LiDFOB lithium salt, the FTIR spectra peaks do not have a significant change; it appears to be the combination of both components. This indicates that the electrolyte components do not undergo spontaneous reactions, remaining stable. The NMR was also performed for the G2 electrolyte and demonstrated in Supporting Information Figure S2 which showed a similar result that the G2 electrolyte appears to be the combination of the GTA with FEC and does not have additional impurity peak or shift of the peak.

The voltage stability of the GTA-based electrolyte was verified by the three-electrode CV scan (Supporting Information Figure S3), the result for the G2 electrolyte is shown in Figure 1c. Three oxidation peaks can be observed on the CV plot which corresponds to the stripping peak of the Li/Li^+^ redox (0.46 V), the stripping peak of the lithium on the platinum (100) plane (0.72 V), and the stripping peak of the lithium on the platinum (110) plane (1.27 V).^33,34^ The stripping peak of the lithium on the platinum (111) plane was not observed in the G1 electrolyte but was observed in the G1 electrolyte at around 1.02 V vs Li/Li^+^. This CV result is also shown to have a high voltage stability window of up to 4.5 V vs Li/Li^+^. The CV scan with the G2 electrolyte (Supporting Information Figure S4) also showed a high voltage stability until 4.2 V without additional redox couples, demonstrating the electrochemical stability of the GTA-based electrolyte when paired with the lithium metal anode. The ionic conductivity of the electrolyte is a crucial factor influencing battery performance. Ionic conductivities of G1, G2, and COM electrolytes were measured by using electrochemical impedance spectroscopy (EIS) with Supporting Information eq S1 and calibrated using a conductivity standard solution. The calculated conductivities are listed in Supporting Information Table S1. The Arrhenius plot in Figure 1d indicates that both G1 and G2 electrolytes exhibit relatively lower ionic conductivities compared to the commercial electrolyte. This difference can be attributed to the higher viscosity of GTA-based electrolytes in comparison to the commercial electrolyte, even though the latter tends to evaporate significantly above 40 °C. The G1 electrolyte also demonstrates higher conductivity than G2, presumably due to its higher lithium salt concentration.

### LFP||Li Half-Cell Cycling at Elevated Temperatures

**Figure 2:**
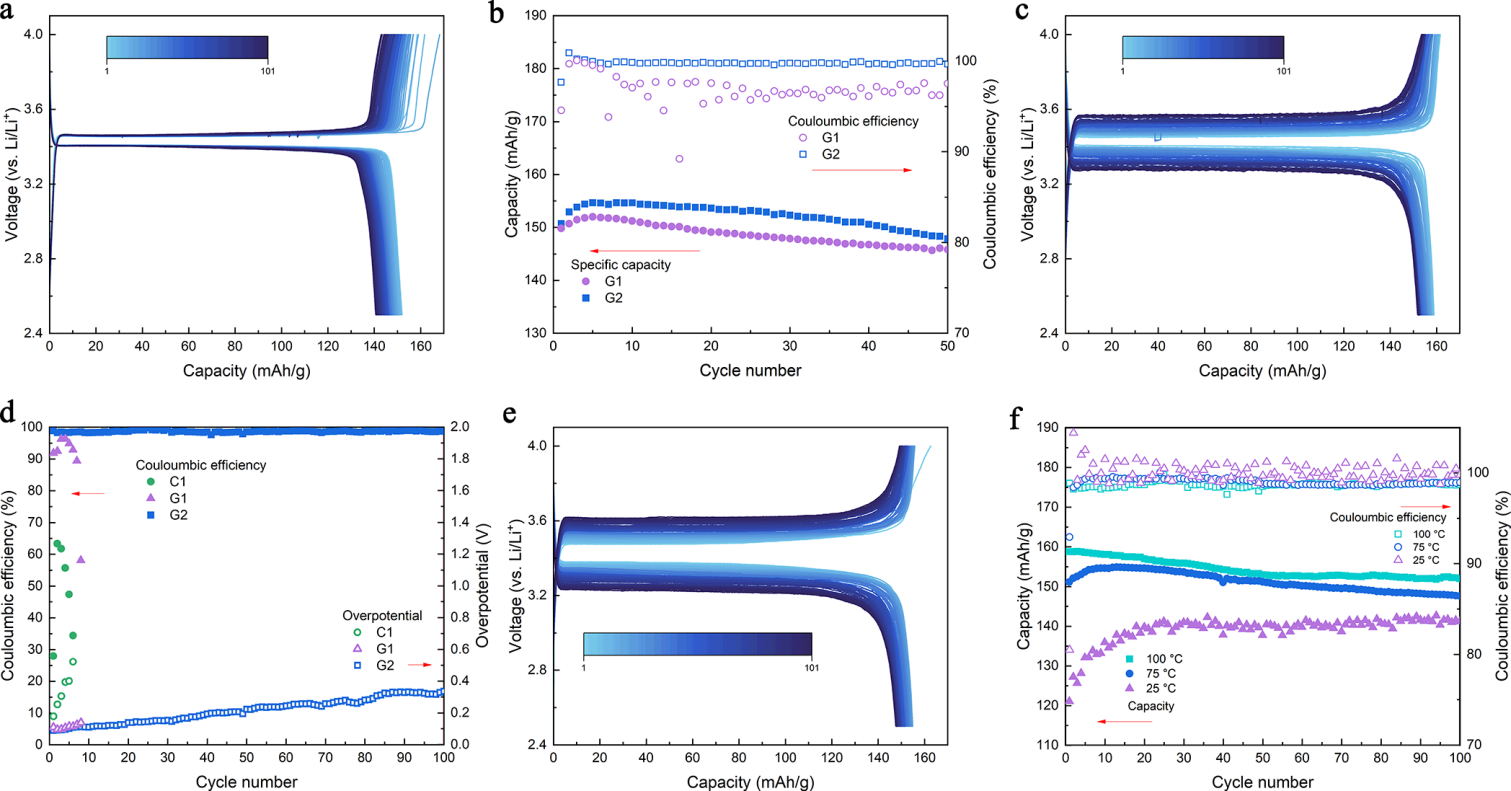
Cycling performance of the Li||LFP half-cell at different temperatures. (a) Charge–discharge curve of the cell using the G1 electrolyte cycled at a 0.1 C rate at 60 °C. (b) Comparison of the capacity retention and CE of the cells using G1 and G2 electrolytes cycled at a 0.1 C rate at 60 °C. (c) Charge–discharge curve of the cell using the G2 electrolyte cycled at a 0.2 C rate at 100 °C. (d) Comparison of the CE and the overpotential evolution during cycling of the cells using COM, G1, and G2 electrolytes cycled at a 0.2 C rate at 100 °C. (e) Charge–discharge curve of the cell using the G2 electrolyte cycled at a 0.2 C rate at 75 °C. (f) Comparison of the capacity retention and CE of the cells using the G2 electrolyte cycled at a 0.2 C rate at 25, 75, and 100 °C.

To demonstrate the capability that the GTA-based electrolyte can be cycled at extreme temperatures, we assembled LFP||Li half-cells with different electrolytes and cycled them at different temperatures. The charge–discharge plot of the half-cell that used G1 electrolyte cycled at 60 °C with a 0.1 C rate is demonstrated in Figure 2a, which showed good stability and low overpotential. A similar performance can be observed for the cell using the G2 electrolyte, which is demonstrated in Supporting Information Figure S5. However, from the capacity retention comparison plot in Figure 2b, we can observe a lower Coulombic efficiency (CE) for the cell using the G1 electrolyte; the average CE for the one used G1 electrolyte is 97.1%, while the one used G2 electrolyte possesses an average CE of 99.7%. The reason for this CE difference is mainly attributed to the addition of FEC and LiDFOB, which helps with the formation of stable CEI and SEI layers that stabilize the electrodes at elevated temperatures. Consequently, a higher specific capacity can also be observed for the one using the G2 electrolyte, which is 147.8 mA h/g while the one using the G1 electrolyte is 145.8 mA h/g after 50 cycles at 60 °C. The room temperature performance of the cells using G1 and G2 electrolytes was also tested. The cell using G1 electrolyte with a 0.2 C rate cycled at 25 °C showed a relatively lower discharge capacity of 111.2 mA h/g at the fifth cycle and a faster capacity decay as we can see from Supporting Information Figure S6. On the other hand, the cell using the G2 electrolyte demonstrated in Supporting Information Figure S7 showed a discharge capacity of 132.1 mA h/g at the fifth cycle and a high discharge capacity of 141.2 mA h/g at the 100th cycle.

At the extreme temperature of 100 °C, the cell using the G1 electrolyte showed only 10 cycles (Supporting Information Figure S8) at a 0.2 C rate before failing. It can be observed that this cell at 100 °C failed with a long charging plateau with a sudden voltage drop, which indicates the dissolution of the CEI layer and is followed by lithium depletion at the cathode side. On the other hand, the cell using the G2 electrolyte showed stable cycling at 100 °C with a capacity retention rate of 95.6%, average CE of 98.6%, and a high cathode specific capacity of 152.0 mA h/g after 100 cycles at 0.2 C rate, as demonstrated in Figure 2c. Similarly, the one using commercial COM electrolyte was also tested at 100 °C at a 0.2 C rate and failed after eight cycles, exhibiting low CE and a noisy charge–discharge curve (Supporting Information Figure S9), indicating a drastic reaction between the cathode with the electrolyte at 100 °C. Although the cell using the G2 electrolyte cycled at 100 °C showed a stable high capacity, it can be observed from the charge–discharge curve that the overpotential of this cell has a significant increase over cycling, indicating that there are still reactions between the electrolyte with the electrode. The EIS of the cell using the G2 electrolyte after cycling showed a large charge-transfer impedance, as demonstrated in Supporting Information Figure S10, indicating that the overpotential increase is mainly due to the Ohmic overpotential increase due to the SEI/CEI layer build-up during cycling at 100 °C. The SEM of the lithium anode after cycling at 100 °C (Supporting Information Figure S11) also supports this conclusion. To quantify this overpotential evaluation of the cells at extreme temperatures, CE and the average overpotential (*V*_ao_) are calculated using Supporting Information eqs S2 and S3.

The calculated results are demonstrated in Figure 2d, and it is obvious that the CE of the GTA-based electrolyte at 100 °C is much higher than that of the one used COM electrolyte, and *V*_ao_ of the GTA-based electrolyte at 100 °C is much lower than that of the COM electrolyte, which proves a superior performance of the cell using the GTA-based electrolyte at higher temperatures. For the COM electrolyte, there will be an irreversible electrolyte decomposition reaction that contributes to the charging capacity, as seen in the charge–discharge curve (Supporting Information Figure 10), and leads to a very low CE with high *V*_ao_ at extreme temperatures. It can also be noticed that the CE of the cell using the G2 electrolyte is higher than the G1 electrolyte. The G1 electrolyte showed a high CE in the first two cycles; however, without filming agents, the stability of the SEI/CEI layer of the electrodes is not good enough to enable stable cycling. The intrinsic instability of the electrodes at high temperatures will dominate when using the G1 electrolyte, the direct reaction between the electrode with the electrolyte consumes the active lithium available and leads to a complete failure of the cell at such a high temperature. The G2 electrolyte which included filming agents along with the high-temperature stable GTA allows the formation of a dense, inorganic-rich CEI/SEI layer and can protect the active material particles and enable a much longer cycle life.

The LFP||Li half-cells with the G2 electrolyte were also tested at an intermediate temperature of 75 °C at a 0.2 C rate, and the charge–discharge plot is demonstrated in Figure 2e. It showed a similar trend with the cell cycled at 100 °C with an increasing overpotential. The comparison of the cell using the G2 electrolyte cycled at different temperatures is demonstrated in Figure 2f, and a higher capacity for the cells cycled at higher temperatures, which could be mainly attributed to the lower impedance at higher temperatures. From the EIS comparison of the cells before cycling at different temperatures (Supporting Information Figure S12), a lower charge-transfer resistance can be observed for the cells at higher temperatures, which is related to the higher ionic conductivity at higher temperatures as demonstrated in Figure 1d. The *V*_ao_ calculated in Supporting Information Figure S13 also showed that the cell cycled at lower temperatures will have a higher overpotential. Moreover, an activation process can be observed on the cells that cycled at lower temperatures, which could be due to sluggish ion transportation at lower temperatures. These results further prove that the G2 electrolyte can enable stable cycling of LFP cells at elevated temperatures.

### Rate Performances at 100 °C in the LFP||Li Half-Cells

**Figure 3:**
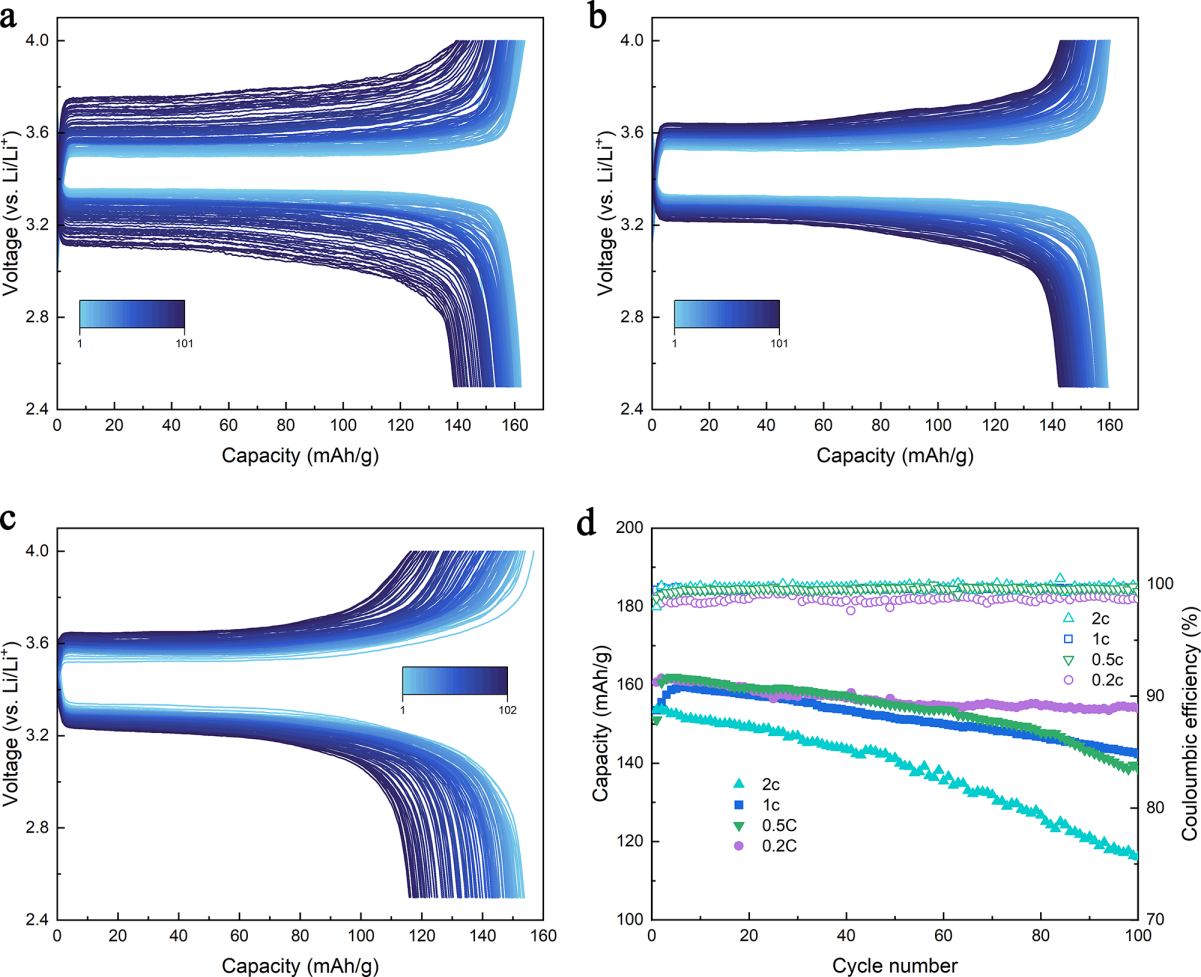
Cycling performance of the Li||LFP half-cell at different C rates at 100 °C. (a) Charge–discharge curve of the cell using the G2 electrolyte cycled at a 0.5 C rate. (b) Charge–discharge curve of the cell using the G2 electrolyte cycled at a 1 C rate. (c) Charge–discharge curve of the cell using the G2 electrolyte cycled at a 2 C rate. (d) Comparison of the capacity retention and CE of the cells using the G2 electrolyte at different C rates.

The rate performance of the LFP||Li half-cell with the G2 electrolyte was also evaluated at 100 °C to demonstrate the kinetics and stability of the cell. At a faster C-rate, the cathode material undergoes a more drastic structural change, resulting in larger overpotential and less distinct plateaus as shown in the charge and discharge curve in Figure 3c (2 C).

In Figure 3a–c, the cells cycled at a faster C-rate demonstrated a larger opening between the charge and discharge curves. The overpotential, as calculated in Supporting Information Figure S14, indicated that the initial overpotential of the cells cycled at a lower C-rate was lower than that of the ones cycled at a higher C-rate, which could be attributed to smaller concentration polarization. However, batteries cycled at different C rates exhibited a similar increase in overpotential, primarily due to the buildup of SEI/CEI, leading to the increased charge-transfer resistance. The capacity retention comparison plot in Figure 3d shows good stability with a high CE for the cells cycled at a high C-rate; the capacity of the cells after 100 cycles at 100 °C with 0.5, 1, and 2 C rates are 138.6, 142.3, and 116.8 mA h/g, respectively. The good capacity retention at high rates observed can be mainly attributed to the improved kinetics at elevated temperatures, where the charge-transfer resistance is much lower than that at lower temperatures.

### NCM523||Li Half-Cell Performance

**Figure 4:**
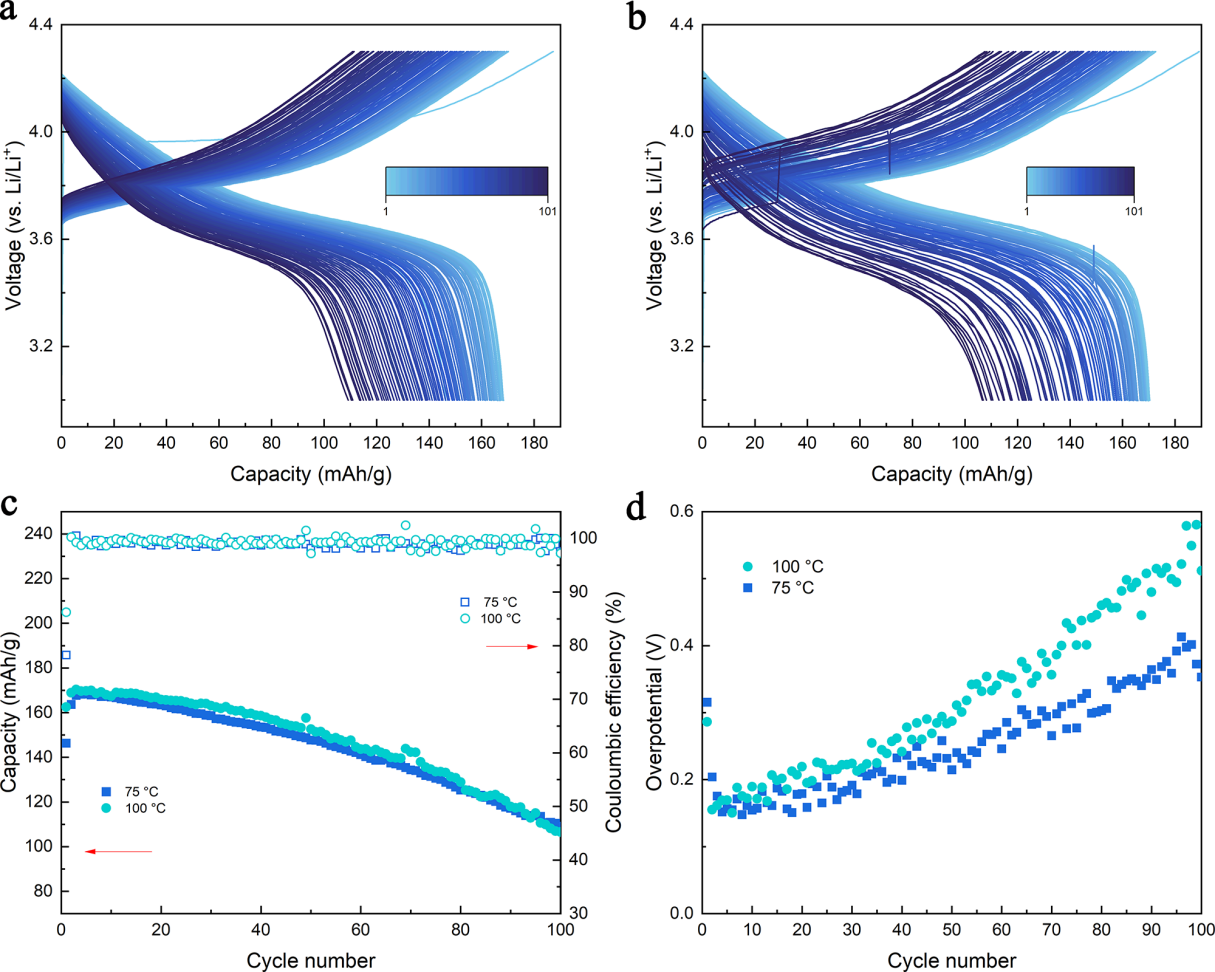
Cycling performance of the Li||NCM523 half-cell at different temperatures. (a) Charge–discharge curve of the cell using the G2 electrolyte cycled at a 1 C rate at 75 °C. (b) Charge–discharge curve of the cell using the G2 electrolyte cycled at a 1 C rate at 100 °C. (c) Capacity retention and CE plot of the cells using the G2 electrolyte cycled at a 1 C rate at different temperatures. (d) Comparison of the overpotential evolution during cycling of the cells using the G2 electrolyte cycled at a 1 C rate at different temperatures.

Besides LFP, half-cells using NCM523 as the cathode material were also tested using the G2 electrolyte to verify the stability of the G2 electrolyte with the high-voltage cathode. Figure 4a shows the charge–discharge curve of the NCM523||Li half-cell with the G2 electrolyte at 75 °C with a 1 C C-rate, which showed good stability for 100 cycles and a capacity of 109.1 mA h/g at the 100th cycle. The cell cycled at 100 °C also showed a similar performance with good stability for 100 cycles, as demonstrated in Figure 4b. From the capacity retention plot in Figure 4c, the NCM523 cell showed a similar decay rate of 66.7 and 65.6% and average CE of 98.9 and 99.2% at 75 and 100 °C respectively, indicating good reversibility for the G2 electrolyte even for the NCM cathode at elevated temperatures. The capacity decay of these NCM523 could mainly result from the instability of the cathode material at higher temperatures, and the surface structure will decompose at elevated temperatures at a higher voltage.^35^ The SEM of the NCM523 cathode after cycling (Supporting Information Figure S15) showed that the cathode material was cracked after 100 cycles at 100 °C at 1 C, which further proves that the cathode failure is the main reason for the capacity decay for NCM chemistry at this temperature. The overpotential of the cell cycled at high temperatures is also compared in Figure 4d, and it is clear that the cell cycled at 100 °C has a much higher overpotential than the cell cycled at 75 °C (0.52 V at 100 °C compared to 0.35 V at 75 °C at the 100th cycle). The higher overpotential at higher temperatures indicates that the SEI/CEI build-up could be the main reason for the capacity fade in the NCM cell, which is different from the LFP cathode we discussed before. Since the NCM particle is less stable than the LFP cathode, especially at high temperatures, the surface of the NCM particle will start to decompose and pulverize, creating a more resistive surface layer with higher overpotential, leading to the complete failure of the battery. TEM imaging in Supporting Information Figure S16 illustrates the NCM cathode material after cycling at 100 °C for 100 cycles. It reveals a CEI layer of 53 nm, significantly thicker than the conventional CEI layer, typically only several nanometers thick. This observation indicates a severe surface reaction on the cathode particles at high temperatures.

Furthermore, the EIS results provided additional insights into the impedance of the NCM after cycling. Supporting Information Figure S17 shows that the overall impedance after cycling was approximately three times higher than the impedance before cycling. Importantly, both the impedance associated with SEI/CEI at higher frequencies and the charge-transfer resistance at lower frequencies increased after cycling. This suggests that in NCM batteries, the decay of the cell performance is not solely attributed to the accumulation of the SEI/CEI layer. It is also influenced by the degradation of the active material particles.

The cycling performance of the commercial electrolyte was also compared with that of the G2 electrolyte at 100 °C. Supporting Information Figure S18 illustrates the comparison between these two systems, demonstrating that the cell using the COM electrolyte exhibits a much faster capacity, and a lower CE can be observed, especially during the first several cycles. This suggests the occurrence of a severe side reaction between the NCM cathode with the commercial electrolyte. Furthermore, Supporting Information Figure S19 demonstrates good stability of the NCM||Li half-cell with G2 electrolyte at lower temperatures at both 25 and 60 °C. However, at 25 °C, the capacity is only about 70% of its theoretical capacity due to the low ionic conductivity and high viscosity. Nevertheless, at 60 °C, an intermediate temperature, the cell exhibits both good capacity and stability with a high CE.

### Flammability Tests in the NCM523||Graphite Pouch Cell

To confirm the stability of the electrolyte during cycling under extreme temperatures, we conducted DSC tests on the G2 and COM electrolytes with the NCM523 cathode (Supporting Information Figure S20). In the COM electrolyte with NCM523, a broad endothermic peak was observed, indicating a continuous reaction process between the COM electrolyte and NCM523 particles. However, no apparent peak was identified in the G2 electrolyte sample, suggesting the good stability of the G2 electrolyte with the NCM523 particles. Moreover, the electrolyte after cycling at 100 °C was collected and tested by NMR and FTIR analysis. Supporting Information Figure S21 demonstrates the NMR spectra of the pristine G2 electrolyte and the G2 electrolyte after cycling in LFP||Li and NCM523||Li half-cells. The spectra indicate that the electrolyte after cycling at 100 °C does not appear to have any additional impurity peak, suggesting that the electrolyte remained stable during the cycling process. However, the NMR peaks corresponding to FEC shifted to a higher chemical shift and with a lower intensity, indicating that FEC was consumed during the cycling. The FTIR spectrum after cycling demonstrated in Supporting Information Figure S22 also supports this observation as no extra impurity peak can be identified in the electrolyte after cycling. However, the peak intensity of the FEC decreased, indicating that FEC may be the component mainly consumed during the cycling of the cell using the G2 electrolyte at high temperatures. In addition, the thermal stability of the G2 electrolyte was also tested by direct exposure to a flame torch and showed a nonflammable property (Supporting Information Figure S23).

**Figure 5:**
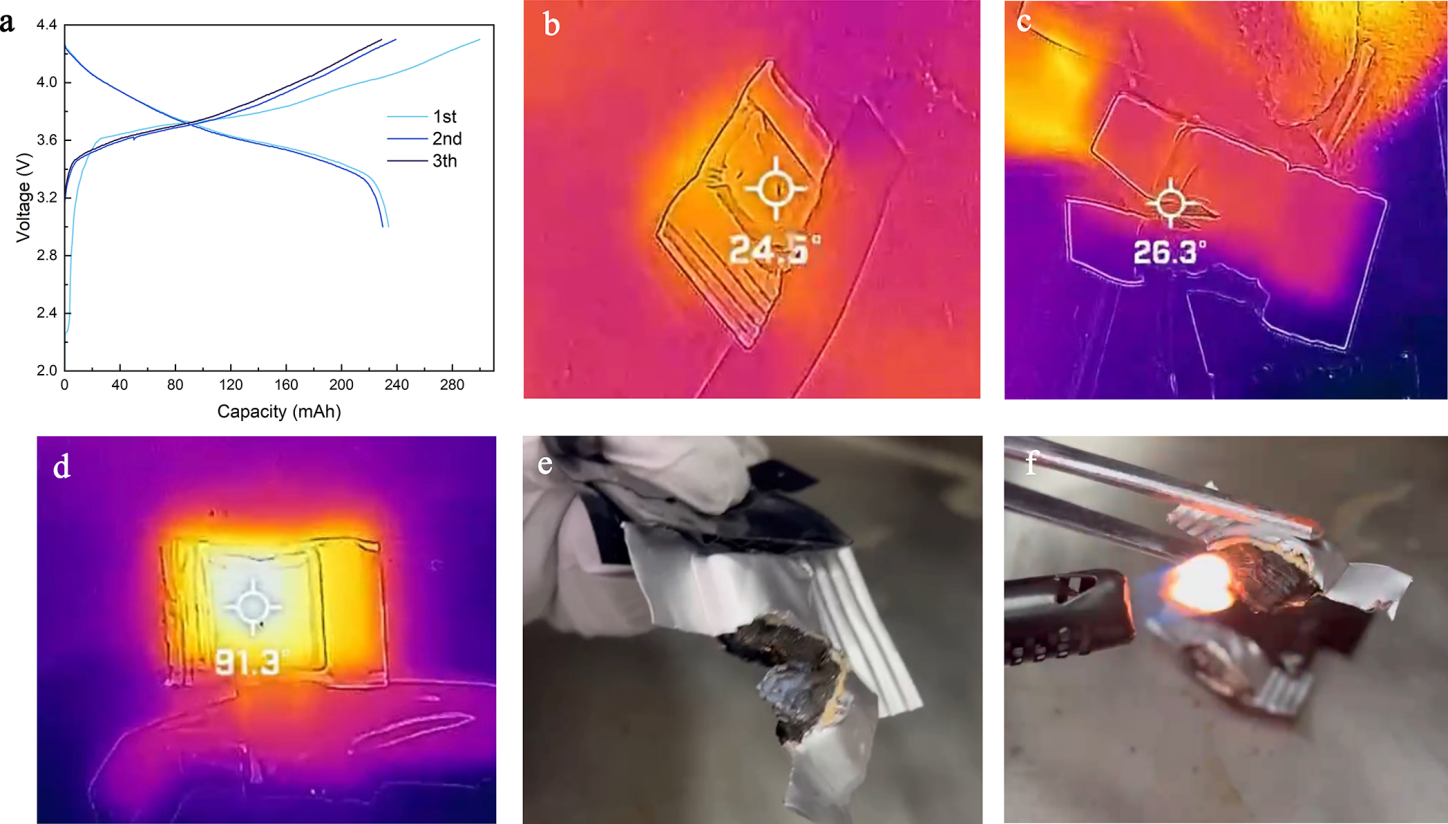
Flammability tests of the NCM523||graphite full pouch cell. (a) Cycling data of the pouch cell using the G2 electrolyte cycled at 100 °C for the flammability tests. (b) Infrared image of the cell using the G2 electrolyte after nail penetration. (c) Infrared image of the cell using the G2 electrolyte after cutting. (d) Infrared image of the cell using the COM electrolyte after nail penetration. (e) Optical image of the cell using the G2 electrolyte after cutting. (f) Optical image of the cell using the G2 electrolyte after cutting and with direct flame contact.

More importantly, the safety properties of the G2 electrolyte were also tested by a nail penetration test with the NCM523||graphite full pouch cell. The pouch cell has a capacity of 250 mA h, which was cycled three times, and ended at a fully charged state at 4.3 V before the nail penetration test, as shown in Figure 5a. The infrared image of the cell after nail penetration using the G2 electrolyte is shown in Figure 5b, which only has a very small temperature increment of 1.5° (room temperature at 23 °C). Even after we cut the pouch cell in half, the temperature increment is very minimal, as shown in Figure 5c. Supporting Information Video 1 also shows that the heat dissipated very fast after cutting, and the temperature went down after only about 30 s.

On the other hand, the cell with the COM electrolyte after nail penetration went up to a much higher temperature (91 °C) within 10 min, as shown in Figure 5d and Supporting Information Video 2. The comparison of the temperature clearly showed that this G2 electrolyte can greatly suppress heat generation when the cell is damaged. With a significantly higher heat capacity (389 J/mol·K) than the conventional electrolyte solvent such as DEC (210 J/mol·K), GTA affording the electrolyte system improved heat tolerance when the cell sustains damage. The high boiling point of the GTA electrolyte also suppresses electrolyte evaporation, thereby avoiding the creation of a local dry zone in the battery. This greatly assists with heat dissipation, precluding propagation of the thermal runaway process.^36^ The temperature increment observed in the cell using the G2 electrolyte was mainly due to the joule heat generated while shorted and dissipated immediately. On the other hand, the one that used COM electrolyte not only had the joule heat but the heat and current also triggered the decomposition of the electrolyte and the SEI layer which contributed mainly to the heat generation during the nail penetration test. The optical image pouch cell with the G2 electrolyte after cutting tests is shown in Figure 5e, which showed no spark or spontaneous ignition during the cutting test. The direct firing test for the cut cell shown in Figure 5f showed no combustion, proving stability and concurrent safety for the G2 electrolyte even with an external heat source.

### Battery Storage Properties in the Li-CF*x* Battery System

The Li-CF*x* battery is a primary battery system with a high energy density. However, several problems limit the widespread use of the Li-CF*x* battery, including the heat generation during the reaction and the low electronic conductivity of the CF*x* particle, which limits the high-rate performance. The crystallization of LiF crystals during the discharging process has a high enthalpy (26.91 kJ mol), resulting in a severe heat generation problem. This exothermic process requires the electrolyte to be capable of operating at high temperatures and preferably nonflammable. Additionally, the irreversible nature of Li-CF*x* chemistry makes it easy to detect any capacity decay during the storage process. A long-term high-temperature storage test was performed on the LFP||Li battery system, as demonstrated in Supporting Information Figure S23. The capacity of the cell after long-term storage was very close to that of the cell only rested for 12 h. Conversely, using Li-CF*x* chemistry makes it easier to detect self-discharge during high-temperature storage. This makes it ideal for studying the long-term storage properties of the GTA-based, high-temperature, flame retardant electrolyte.

**Figure 6:**
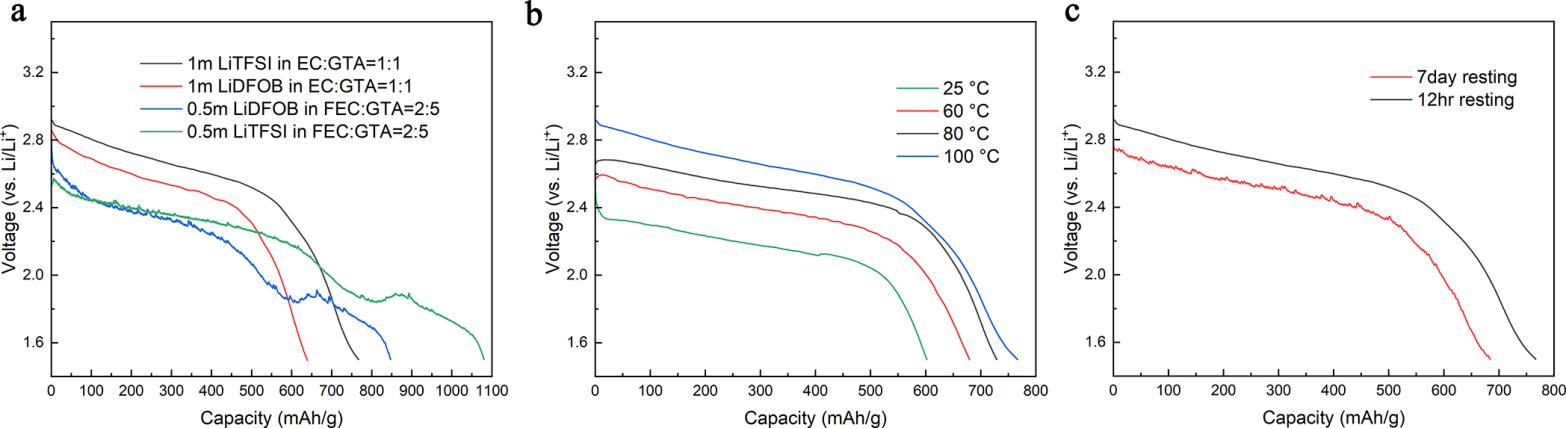
Li||CF*x* half-cell performances. (a) Discharge curve of the Li-CF*x* cell using different electrolyte systems at 100 °C, and the C-rate is 0.05 C. (b) Discharge curve of the Li-CF*x* cell using the G1 electrolyte at different temperatures. (c) Discharge curve of the Li-CF*x* cell using the G1 electrolyte after different resting times at 100 °C.

The stability of the Li-CF*x* battery chemistry with different GTA-based electrolyte systems is studied first, and the results are shown in Figure 6a. Both cells using the FEC-containing electrolyte exhibited a lower discharge voltage and a higher discharge capacity with two plateaus. However, the cell with the EC containing an electrolyte does not have that behavior. The unusual result that we observed in the FEC-containing systems could come from the reaction between the CF*x* electrode and the FEC electrolyte. It is believed that the discharge process of the CF*x* cathode includes a step, in which the electrolyte intercalates into the layered structure of the CF*x*. As a result, the fluorine in FEC may be more reactive than the fluorine in CF*x*, which lowers the discharge voltage and contributes to the capacity. Thus, we used electrolyte G1, which does not include FEC and demonstrated the best capacity as the electrolyte for our future studies of the Li-CF*x* battery system.

Similar to LFP and NCM, the Li-CF*x* battery with the GTA-based electrolyte also showed a relatively lower capacity of 602 mA h/g at room temperature. However, the capacity of the Li-CF*x* battery above becomes higher as the temperature increases and becomes closer to its theoretical capacity of 865 mA h/g. Nevertheless, the discharge profile in Figure 6b demonstrates that discharge voltage is still noticeably lower at lower temperatures due to lower ionic conductivity. The discharge curve of the battery using the G1 electrolyte stored at 100 °C for 7 days is presented in Figure 6c, and the battery showed a 9% decay after 7 days (692 mA h/g after 7 days resting) which is lower than the one using the COM electrolyte at 100 °C (13% decay after 7 days at 100 °C), as shown in Supporting Information Figure S25. These results demonstrate that GTA-based flame-retardant electrolyte not only enables a high-temperature stable Li-CF*x* battery but also allows the battery to be stored at a very high temperature without significant capacity decay over time, which could be promising for specialized applications such as space missions.

## Conclusions

In this work, we introduced GTA as a high-temperature electrolyte solvent that is stable and nonflammable under a range of conditions. We evaluated the performance of GTA-based electrolytes in three different battery chemistries, LFP, NCM523, and CF*x* under extreme temperatures and demonstrated their stability and functionality at high temperatures. Furthermore, the high-temperature decay mechanism of LFP and NCM523 cathode materials was also investigated. For LFP batteries, we identified the buildup of the SEI/CEI layer as the primary cause of the capacity decay and increased overpotential. On the other hand, for NCM523 batteries, the spontaneous structural instability at the surface of the cathode material was found to also contribute to capacity decay. Additionally, Li-CF*x* electrodes were used to assess the high-temperature storage stability of the GTA-based electrolyte. A comparison of the GTA-based high-temperature electrolyte system with other previously reported high-temperature electrolyte systems is detailed in Supporting Information Table 2. As can be concluded from the data, the GTA-based system clearly demonstrates exceptional performance at high temperatures compared to that of other electrolyte systems. Overall, GTA as a low-cost and readily available high-temperature nonflammable electrolyte holds promise for adoption in current lithium-ion battery production, could provide the battery with better safety, and creates opportunities for high-temperature applications.

## Data Availability

The data that support the findings of this study are available upon reasonable request.

## Supporting Information Available

TGA results of G1, G2, and COM electrolyte; H^1^ NMR spectrum of the G2 electrolyte and GTA and FEC; image of the three-electrode setup that we used in our experiment; CV scan of the G1 electrolyte; charge–discharge curve of the half-cells using G1, G2, and COM electrolytes with the LFP cathode at different temperatures; EIS measurement and SEM image of the LFP half-cell after cycling; EIS of the LFP half-cells at different temperatures; overpotential evolution of the LFP half-cells at different temperatures; overpotential evolution of the LFP half-cells at different C rates cycled at 100 °C; SEM of the NCM523 cathode after cycling at 100 °C; TEM image of the NCM523 cathode after cycling at 1 C at 100 °C; EIS of the NCM523 half-cells before and after cycling; capacity retention and CE of the NCM523 half-cells using G2 and COM electrolytes at 100 °C; capacity retention and CE of the NCM523 half-cells using G2 at different temperatures; DSC results of the COM and G2 electrolyte with the NCM523 cathode material; ^1^H NMR spectrum of the G2 electrolyte before cycling and after cycling; FTIR spectrum of the G2 electrolyte before cycling and after cycling; direct firing test of the G2 electrolyte with the torch; first three charge–discharge curve of the LFP||Li cell using the G2 electrolyte after different resting times at 100 C; discharge curve of the Li-CF*x* cell after long-term resting using the COM electrolyte; calculated conductivity of the G1, G2, and commercial electrolyte at different temperatures; and comparison of our results with different reported high-temperature electrolyte systems (PDF)
Nail penetration test on the pouch cells using G2 electrolyte (MP4)
Nail penetration test on the pouch cells using COM electrolyte (MP4)

## References

[ref1] Stampatori D.; Raimondi P. P.; Noussan M. Li-Ion Batteries: A Review of a Key Technology for Transport Decarbonization. Energies 2020, 13 (10), 2638 10.3390/en13102638.

[ref2] Xu C.; Dai Q.; Gaines L.; Hu M.; Tukker A.; Steubing B. Future Material Demand for Automotive Lithium-Based Batteries. Commun. Mater. 2020, 1, 99 10.1038/s43246-020-00095-x.

[ref3] Hess S.; Wohlfahrt-Mehrens M.; Wachtler M. Flammability of Li-Ion Battery Electrolytes: Flash Point and Self-Extinguishing Time Measurements. J. Electrochem. Soc. 2015, 162 (2), A3084–A3097. 10.1149/2.0121502jes.

[ref4] Ren D.; Feng X.; Liu L.; Hsu H.; Lu L.; Wang L.; He X.; Ouyang M. Investigating the Relationship between Internal Short Circuit and Thermal Runaway of Lithium-Ion Batteries under Thermal Abuse Condition. Energy Storage Mater. 2021, 34, 563–573. 10.1016/j.ensm.2020.10.020.

[ref5] Meng L.; Wang G.; See K. W.; Wang Y.; Zhang Y.; Zang C.; Zhou R.; Xie B. Large-Scale Li-Ion Battery Research and Application in Mining Industry. Energies 2022, 15, 3884 10.3390/en15113884.

[ref6] Ma S.; Jiang M.; Tao P.; Song C.; Wu J.; Wang J.; Deng T.; Shang W. Temperature Effect and Thermal Impact in Lithium-Ion Batteries: A Review. Prog. Nat. Sci. 2018, 28 (6), 653–666. 10.1016/j.pnsc.2018.11.002.

[ref7] Chen T.; Jin Z.; Liu Y.; Zhang X.; Wu H.; Li M.; Feng W. W.; Zhang Q.; Wang C. Stable High-Temperature Lithium-Metal Batteries Enabled by Strong Multiple Ion-Dipole Interactions. Angew. Chem., Int. Ed. 2022, 61 (35), e202207645 10.1002/anie.202207645. 35793172

[ref8] Fan X.; Chen L.; Borodin O.; Ji X.; Chen J.; Hou S.; Deng T.; Zheng J.; Yang C.; Liou S. C.; Amine K.; Xu K.; Wang C. Non-Flammable Electrolyte Enables Li-Metal Batteries with Aggressive Cathode Chemistries. Nat. Nanotechnol. 2018, 13, 715–722. 10.1038/s41565-018-0183-2. 30013215

[ref9] Zeng Z.; Murugesan V.; Han K. S.; Jiang X.; Cao Y.; Xiao L.; Ai X.; Yang H.; Zhang J. G.; Sushko M. L.; Liu J. Non-Flammable Electrolytes with High Salt-to-Solvent Ratios for Li-Ion and Li-Metal Batteries. Nat. Energy 2018, 3, 674–681. 10.1038/s41560-018-0196-y.

[ref10] Yim T.; Park M. S.; Woo S. G.; Kwon H. K.; Yoo J. K.; Jung Y. S.; Kim K. J.; Yu J. S.; Kim Y. J. Self-Extinguishing Lithium Ion Batteries Based on Internally Embedded Fire-Extinguishing Microcapsules with Temperature-Responsiveness. Nano Lett. 2015, 15 (8), 5059–5067. 10.1021/acs.nanolett.5b01167. 26177284

[ref11] Doi T.; Taccori R. J.; Fujii R.; Nagashima T.; Endo T.; Kimura Y.; Inaba M. Non-Flammable and Highly Concentrated Carbonate Ester-Free Electrolyte Solutions for 5 V-Class Positive Electrodes in Lithium-Ion Batteries. ChemSusChem 2021, 14, 2445–2451. 10.1002/cssc.202100523. 33961342

[ref12] Wang J.; Yamada Y.; Sodeyama K.; Watanabe E.; Takada K.; Tateyama Y.; Yamada A. Fire-Extinguishing Organic Electrolytes for Safe Batteries. Nat. Energy 2017, 3, 22–29. 10.1038/s41560-017-0033-8.

[ref13] Gond R.; Van Ekeren W.; Mogensen R.; Naylor A. J.; Younesi R. Non-Flammable Liquid Electrolytes for Safe Batteries. Mater. Horiz. 2021, 8, 2913–2928. 10.1039/D1MH00748C. 34549211

[ref14] Shiga T.; Kato Y.; Kondo H.; Okuda C. A. Self-Extinguishing Electrolytes Using Fluorinated Alkyl Phosphates for Lithium Batteries. J. Mater. Chem. A 2017, 5, 5156–5162. 10.1039/C6TA09915G.

[ref15] Albertus P.; Anandan V.; Ban C.; Balsara N.; Belharouak I.; Buettner-Garrett J.; Chen Z.; Daniel C.; Doeff M.; Dudney N. J.; Dunn B.; Harris S. J.; Herle S.; Herbert E.; Kalnaus S.; Libera J. A.; Lu D.; Martin S.; McCloskey B. D.; McDowell M. T.; Meng Y. S.; Nanda J.; Sakamoto J.; Self E. C.; Tepavcevic S.; Wachsman E.; Wang C.; Westover A. S.; Xiao J.; Yersak T. Challenges for and Pathways toward Li-Metal-Based All-Solid-State Batteries. ACS Energy Lett. 2021, 6, 1399–1404. 10.1021/acsenergylett.1c00445.

[ref16] Shen W.; Wang N.; Zhang J.; Wang F.; Zhang G. Heat Generation and Degradation Mechanism of Lithium-Ion Batteries during High-Temperature Aging. ACS Omega 2022, 7, 44733–44742. 10.1021/acsomega.2c04093. 36530310 PMC9753165

[ref17] Parimalam B. S.; MacIntosh A. D.; Kadam R.; Lucht B. L. Decomposition Reactions of Anode Solid Electrolyte Interphase (SEI) Components with LiPF_6_. J. Phys. Chem. C 2017, 121 (41), 22733–22738. 10.1021/acs.jpcc.7b08433.

[ref18] Bravo Diaz L.; He X.; Hu Z.; Restuccia F.; Marinescu M.; Barreras J. V.; Patel Y.; Offer G.; Rein G. Review—Meta-Review of Fire Safety of Lithium-Ion Batteries: Industry Challenges and Research Contributions. J. Electrochem. Soc. 2020, 167 (9), 090559 10.1149/1945-7111/aba8b9.

[ref19] Edge J. S.; O’Kane S.; Prosser R.; Kirkaldy N. D.; Patel A. N.; Hales A.; Ghosh A.; Ai W.; Chen J.; Yang J.; Li S.; Pang M. C.; Bravo Diaz L.; Tomaszewska A.; Marzook M. W.; Radhakrishnan K. N.; Wang H.; Patel Y.; Wu B.; Offer G. J. Lithium Ion Battery Degradation: What You Need to Know. Phys. Chem. Chem. Phys. 2021, 23, 8200–8221. 10.1039/D1CP00359C. 33875989

[ref20] Nagarajan S.; Weiland C.; Hwang S.; Balasubramanian M.; Arava L. M. R. Depth-Dependent Understanding of Cathode Electrolyte Interphase (CEI) on the Layered Li-Ion Cathodes Operated at Extreme High Temperature. Chem. Mater. 2022, 34, 4587–4601. 10.1021/acs.chemmater.2c00435.

[ref21] Hou J.; Yang M.; Wang D.; Zhang J. Fundamentals and Challenges of Lithium Ion Batteries at Temperatures between −40 and 60 °C. Adv. Energy Mater. 2020, 10, 1904152 10.1002/aenm.201904152.

[ref22] Zhan C.; Wu T.; Lu J.; Amine K. Dissolution, Migration, and Deposition of Transition Metal Ions in Li-Ion Batteries Exemplified by Mn-Based Cathodes-A Critical Review. Energy Environ. Sci. 2018, 11, 243–257. 10.1039/C7EE03122J.

[ref23] Jayasree S. S.; Murali A. S.; Nair S.; Santhanagopalan D. Recent Progress on the Low and High Temperature Performance of Nanoscale Engineered Li-Ion Battery Cathode Materials. Nanotechnology 2022, 33, 352001 10.1088/1361-6528/ac67ac. 35428032

[ref24] Utsunomiya T.; Hatozaki O.; Yoshimoto N.; Egashira M.; Morita M. Self-Discharge Behavior and Its Temperature Dependence of Carbon Electrodes in Lithium-Ion Batteries. J. Power Sources 2011, 196 (20), 8598–8603. 10.1016/j.jpowsour.2011.05.066.

[ref25] Chen L.; Wu H.; Ai X.; Cao Y.; Chen Z. Toward Wide temperature Electrolyte for Lithium-Ion Batteries. Battery Energy 2022, 1, 20210006 10.1002/bte2.20210006.

[ref26] Kohlmeyer R. R.; Horrocks G. A.; Blake A. J.; Yu Z.; Maruyama B.; Huang H.; Durstock M. F. Pushing the Thermal Limits of Li-Ion Batteries. Nano Energy 2019, 64, 103927 10.1016/j.nanoen.2019.103927.

[ref27] Sloop S. E.; Kerr J. B.; Kinoshita K. The Role of Li-Ion Battery Electrolyte Reactivity in Performance Decline and Self-Discharge. J. Power Sources 2003, 119–121, 330–337. 10.1016/S0378-7753(03)00149-6.

[ref28] Leng F.; Tan C. M.; Pecht M. Effect of Temperature on the Aging Rate of Li Ion Battery Operating above Room Temperature. Sci. Rep. 2015, 5, 12967 10.1038/srep12967. 26245922 PMC4526891

[ref29] Grünebaum M.; Buchheit A.; Lürenbaum C.; Winter M.; Wiemhöfer H. D. Ester-Based Battery Solvents in Contact with Metallic Lithium: Effect of Water and Alcohol Impurities. J. Phys. Chem. C 2019, 123 (12), 7033–7044. 10.1021/acs.jpcc.9b00331.

[ref30] Hou T.; Yang G.; Rajput N. N.; Self J.; Park S. W.; Nanda J.; Persson K. A. The Influence of FEC on the Solvation Structure and Reduction Reaction of LiPF_6_/EC Electrolytes and Its Implication for Solid Electrolyte Interphase Formation. Nano Energy 2019, 64, 103881 10.1016/j.nanoen.2019.103881.

[ref31] Brown Z. L.; Jurng S.; Nguyen C. C.; Lucht B. L. Effect of Fluoroethylene Carbonate Electrolytes on the Nanostructure of the Solid Electrolyte Interphase and Performance of Lithium Metal Anodes. ACS Appl. Energy Mater. 2018, 1 (7), 3057–3062. 10.1021/acsaem.8b00705.

[ref32] Dong Q.; Guo F.; Cheng Z.; Mao Y.; Huang R.; Li F.; Dong H.; Zhang Q.; Li W.; Chen H.; Luo Z.; Shen Y.; Wu X.; Chen L. Insights into the Dual Role of Lithium Difluoro(Oxalato)Borate Additive in Improving the Electrochemical Performance of NMC811||Graphite Cells. ACS Appl. Energy Mater. 2020, 3 (1), 695–704. 10.1021/acsaem.9b01894.

[ref33] Aurbach D.; Daroux M.; Faguy P.; Yeager E. The Electrochemistry of Noble Metal Electrodes in Aprotic Organic Solvents Containing Lithium Salts. J. Electroanal. Chem. 1991, 297 (1), 225–244. 10.1016/0022-0728(91)85370-5.

[ref34] Moshkovich M.; Gofer Y.; Aurbach D. Investigation of the Electrochemical Windows of Aprotic Alkali Metal (Li, Na, K) Salt Solutions. J. Electrochem. Soc. 2001, 148, E155 10.1149/1.1357316.

[ref35] Li J.; Hua H.; Kong X.; Yang H.; Dai P.; Zeng J.; Zhao J. In-Situ Probing the near-Surface Structural Thermal Stability of High-Nickel Layered Cathode Materials. Energy Storage Mater. 2022, 46, 90–99. 10.1016/j.ensm.2022.01.007.

[ref36] Feng X.; Ouyang M.; Liu X.; Lu L.; Xia Y.; He X. Thermal Runaway Mechanism of Lithium Ion Battery for Electric Vehicles: A Review. Energy Storage Mater. 2018, 10, 246–267. 10.1016/j.ensm.2017.05.013.

