# Supplementary material for: Glycerol Triacetate-Based Flame Retardant High-Temperature Electrolyte for the Lithium-Ion Battery
Source: ACS Appl Mater Interfaces. 2024 May 6;16(19):24590–600. doi: 10.1021/acsami.4c02323 (PMC11103651; doi:10.1021/acsami.4c02323)
Supplement: Supplementary file 1 — am4c02323_si_001.pdf [file am4c02323_si_001.pdf]

Supporting information

A Glycerol triacetate based flame retardant high-temperature electrolyte for the lithium ion battery

Xinsheng Wu[a], Tong Liu[b], Young-Geun Lee[a] and Jay. F. Whitacre[a,c*]

[a] *Department of Materials Science and Engineering, Carnegie Mellon University, 5000 Forbes Avenue, Pittsburgh, Pennsylvania, 15213, USA*
[b] *Department of Chemistry, Carnegie Mellon University, 4400 Fifth Avenue, Pittsburgh, Pennsylvania, 15213, USA*
[c] *Scott Institute for Energy Innovation, Carnegie Mellon University, 5000 Forbes Avenue, Pittsburgh, Pennsylvania, 15213, USA*

corresponding author's email address: whitacre@andrew.cmu.edu

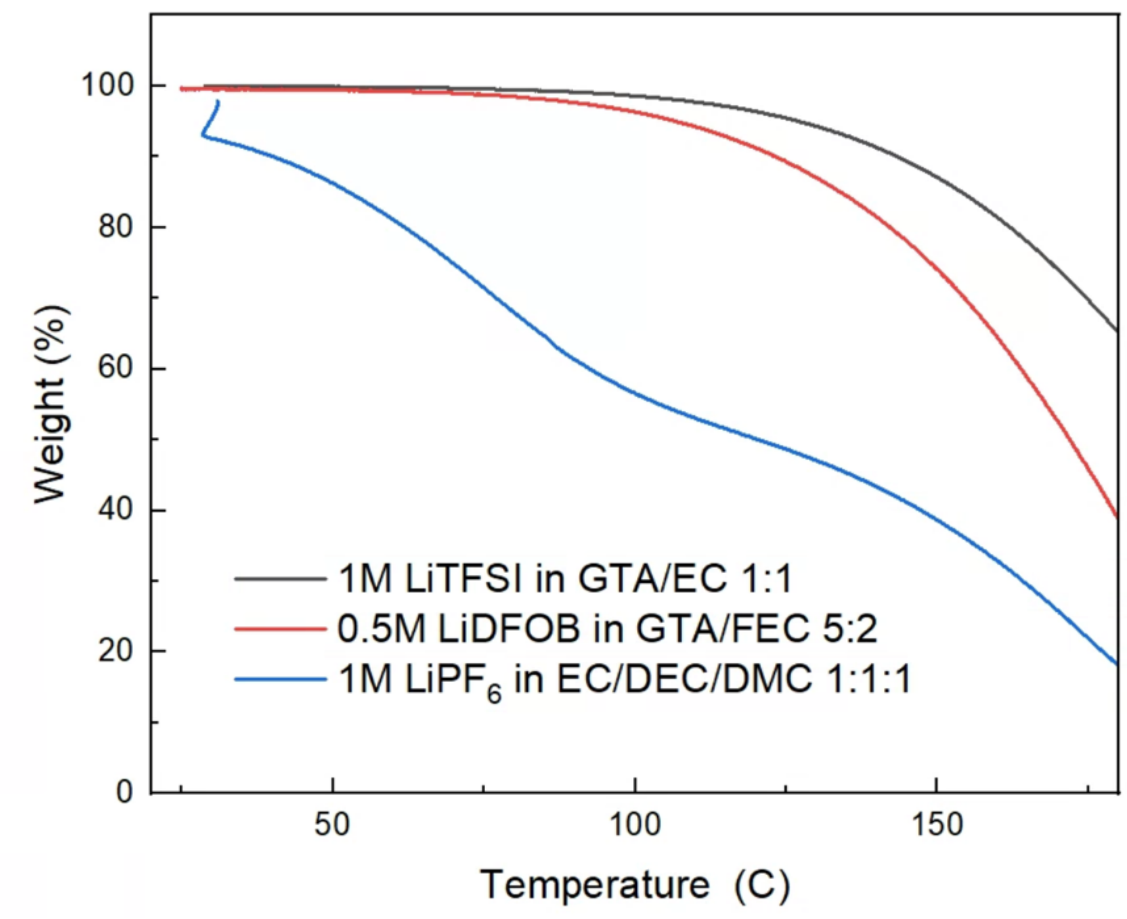

Figure S1. TGA results of G1, G2 and COM electrolyte.

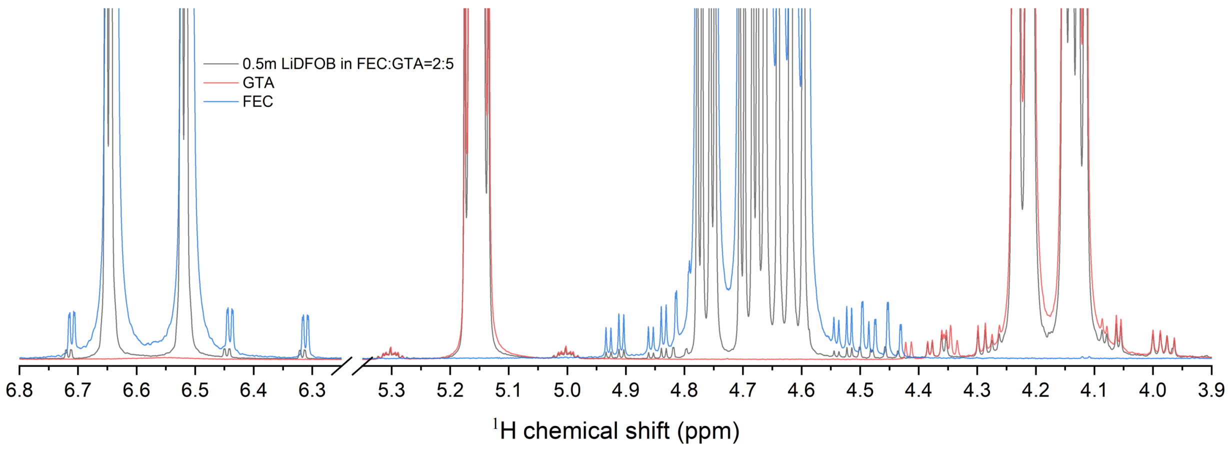

Figure S2. H^1 NMR spectrum of the G2 electrolyte and GTA and FEC

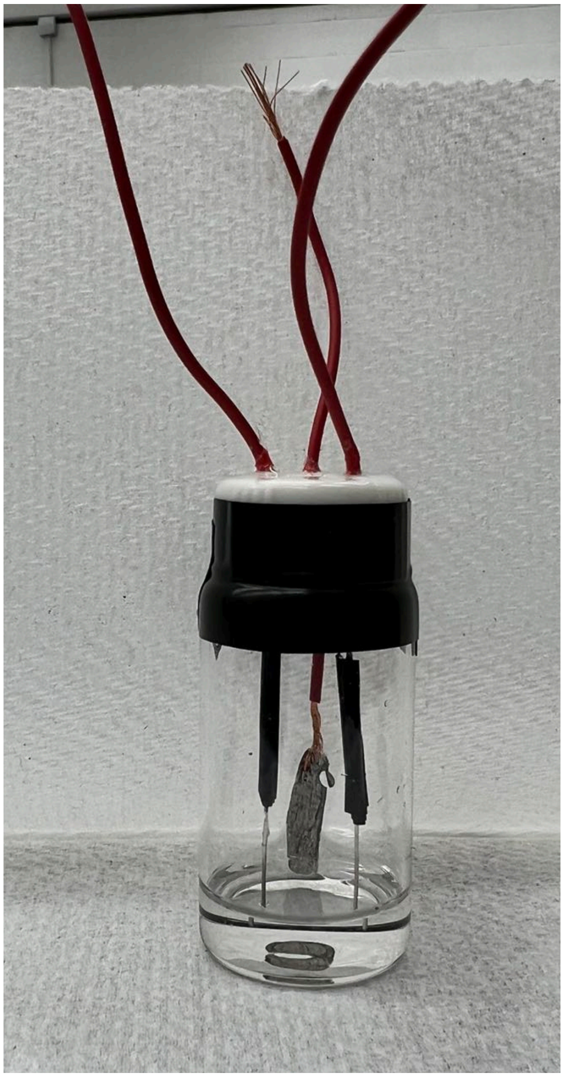

Figure S3. Image of the three-electrode setup that we used in our experiment.

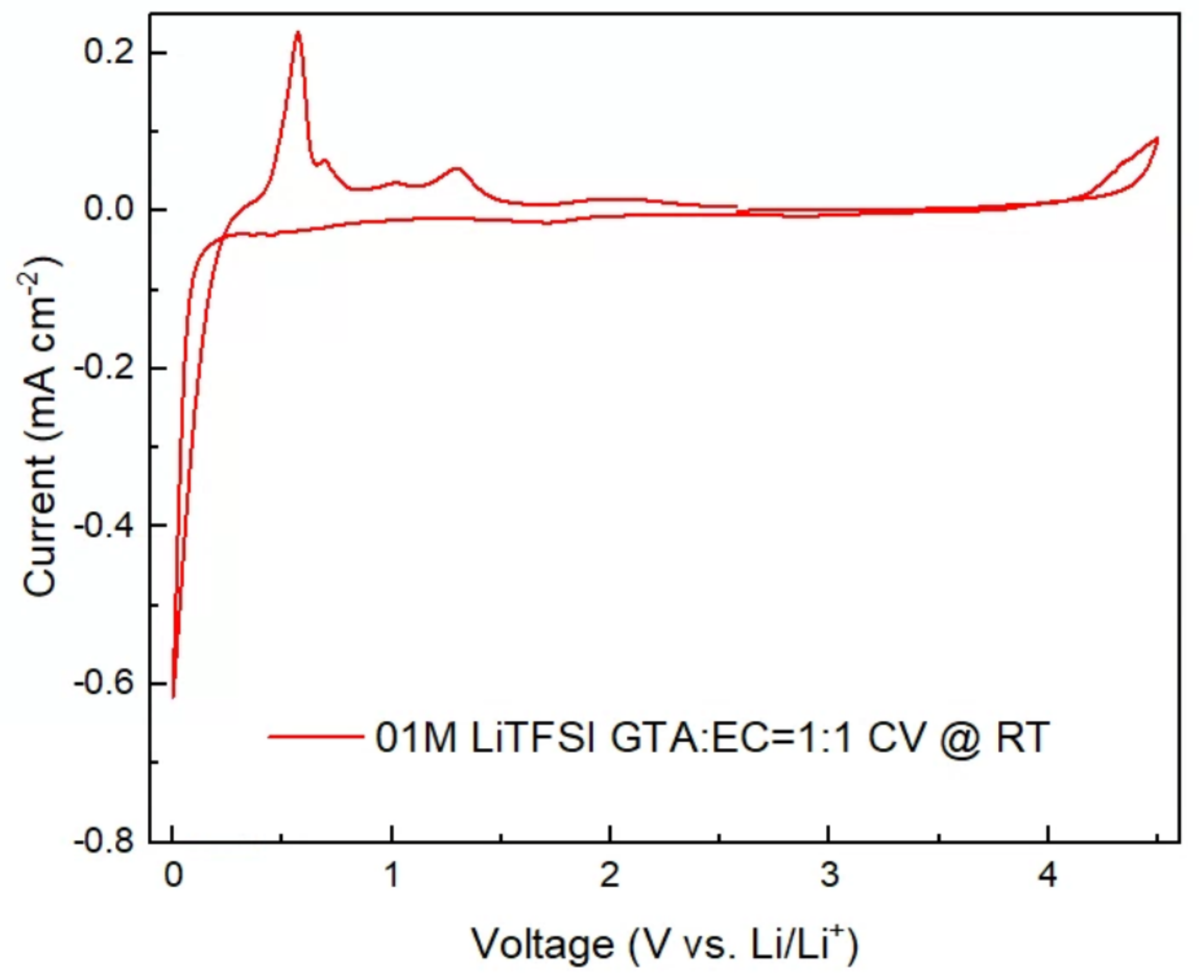

Figure S4. CV scan of the G1 electrolyte in a three electrode cell.

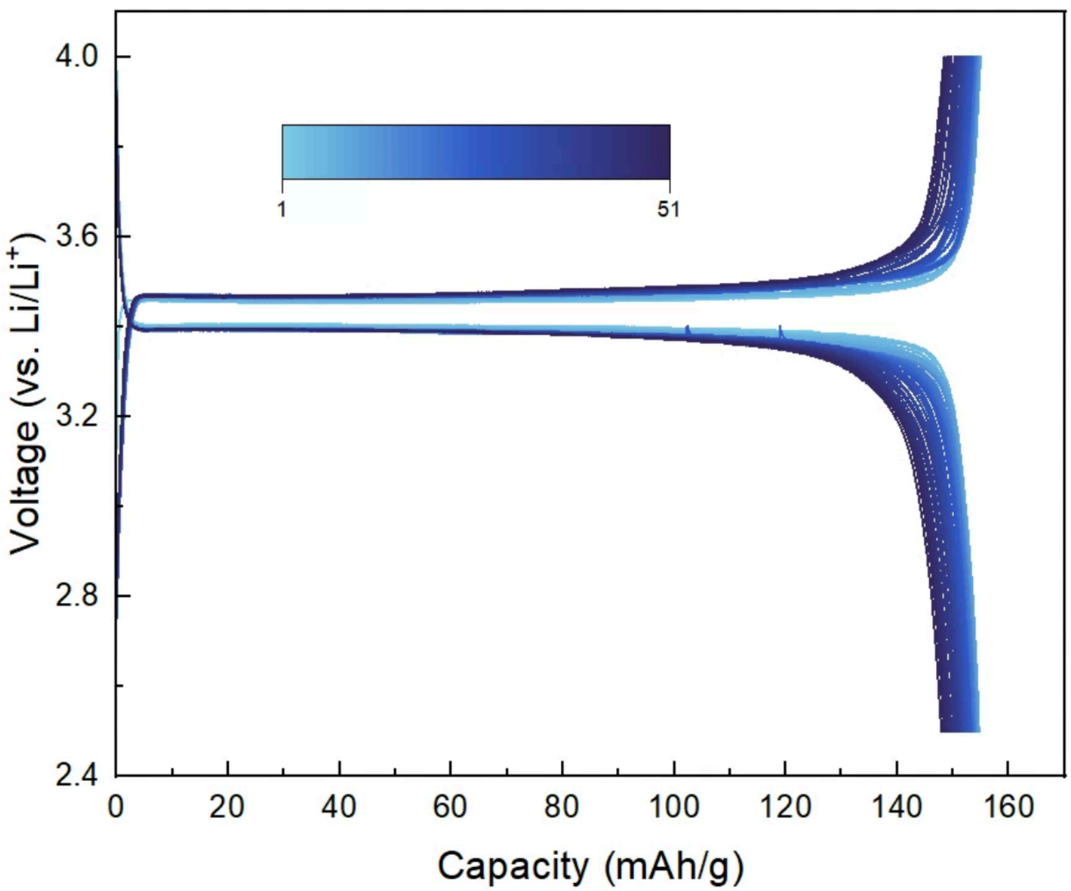

Figure S5. Charge-discharge curve of the LFP||Li cell using G2 electrolyte cycled at a 0.1C rate at 60 °C

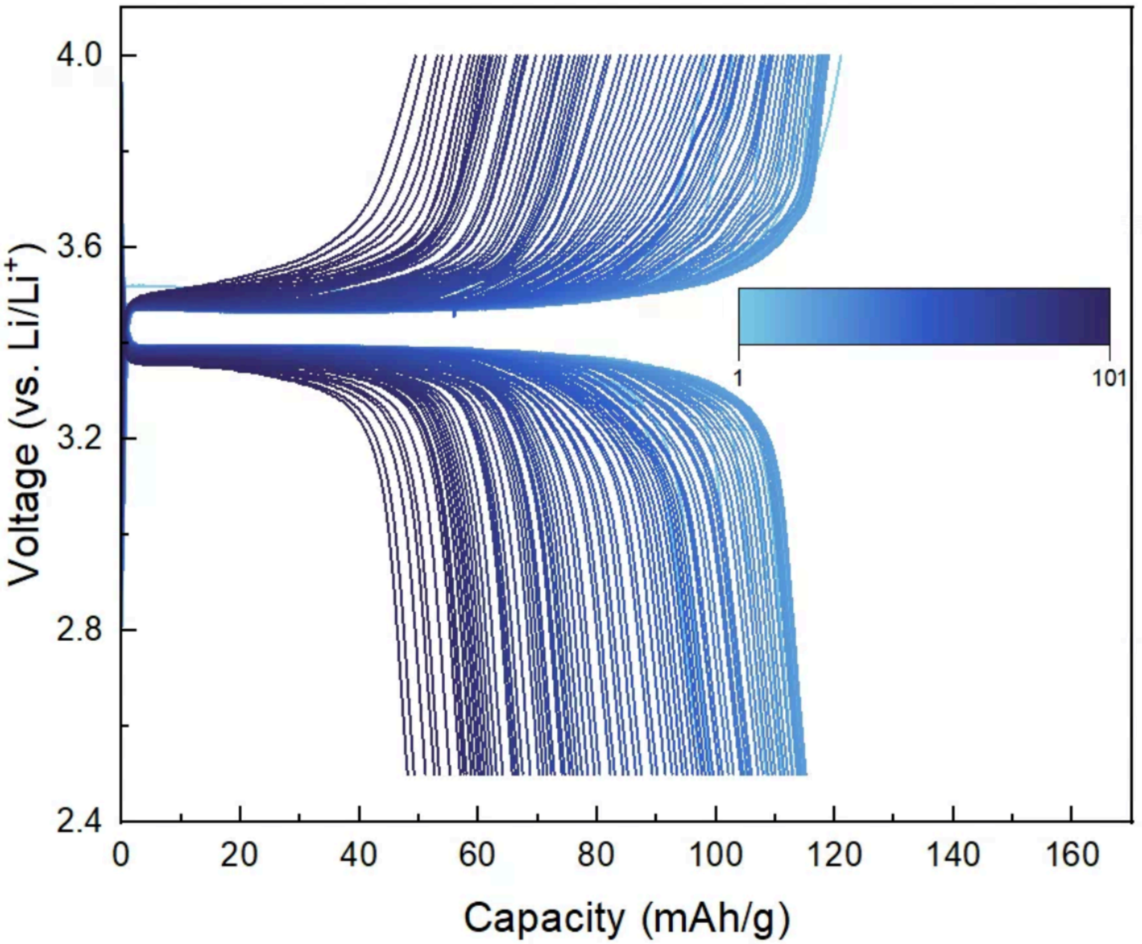

Figure S6. Charge-discharge curve of the LFP||Li cell using G1 electrolyte cycled at a 0.2C rate at 25 °C

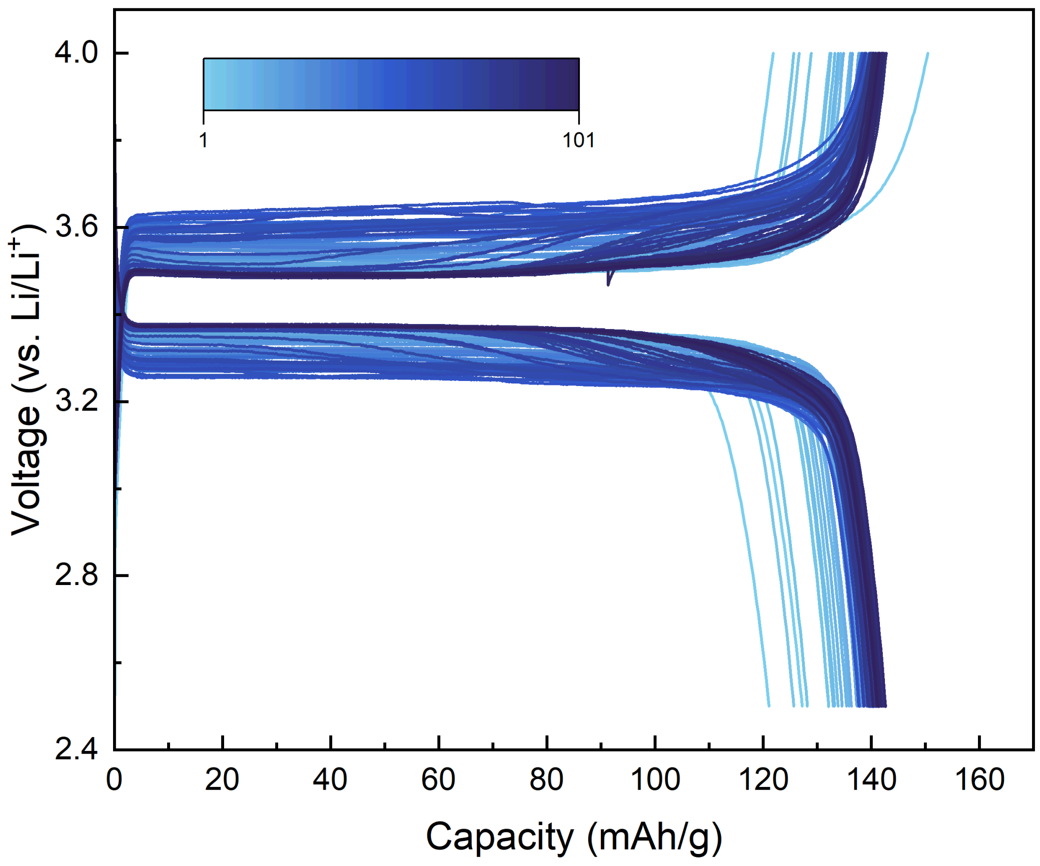

Figure S7. Charge-discharge curve of the LFP||Li cell using G2 electrolyte cycled at a 0.2C rate at 25 °C

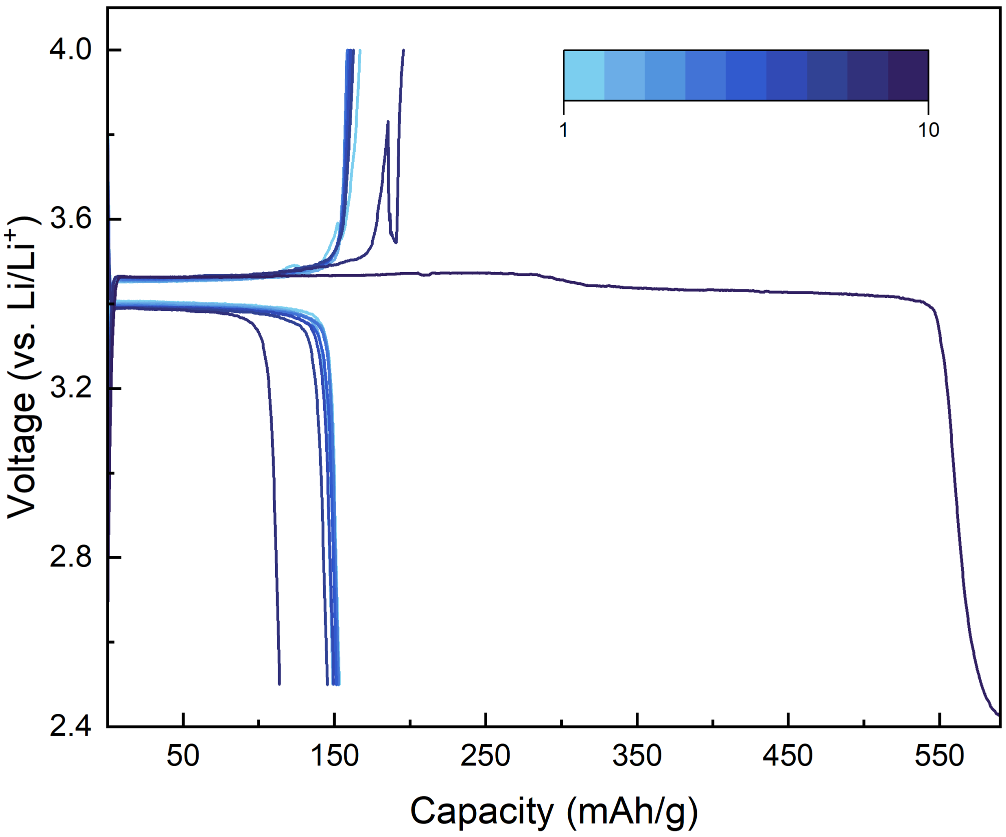

Figure S8. Charge-discharge curve of the LFP||Li cell using G1 electrolyte cycled at a 0.2C rate at 100 °C

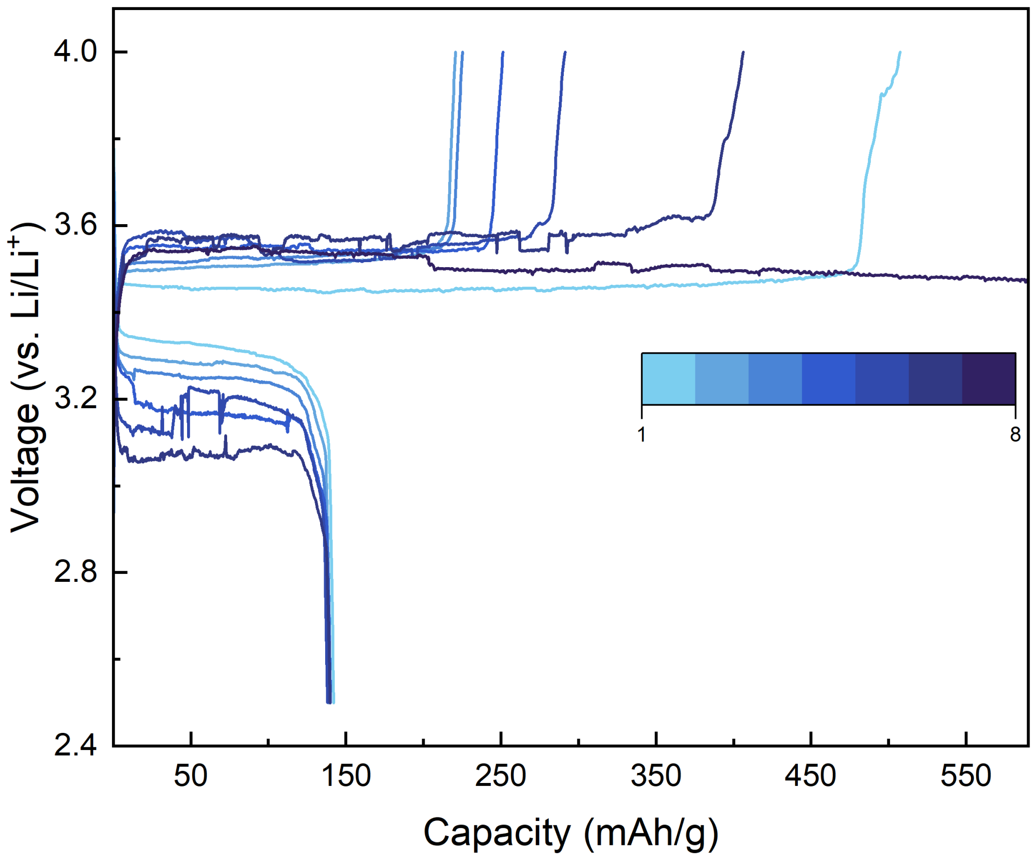

Figure S9. Charge-discharge curve of the LFP||Li cell using COM electrolyte cycled at a 0.2C rate at 100 °C

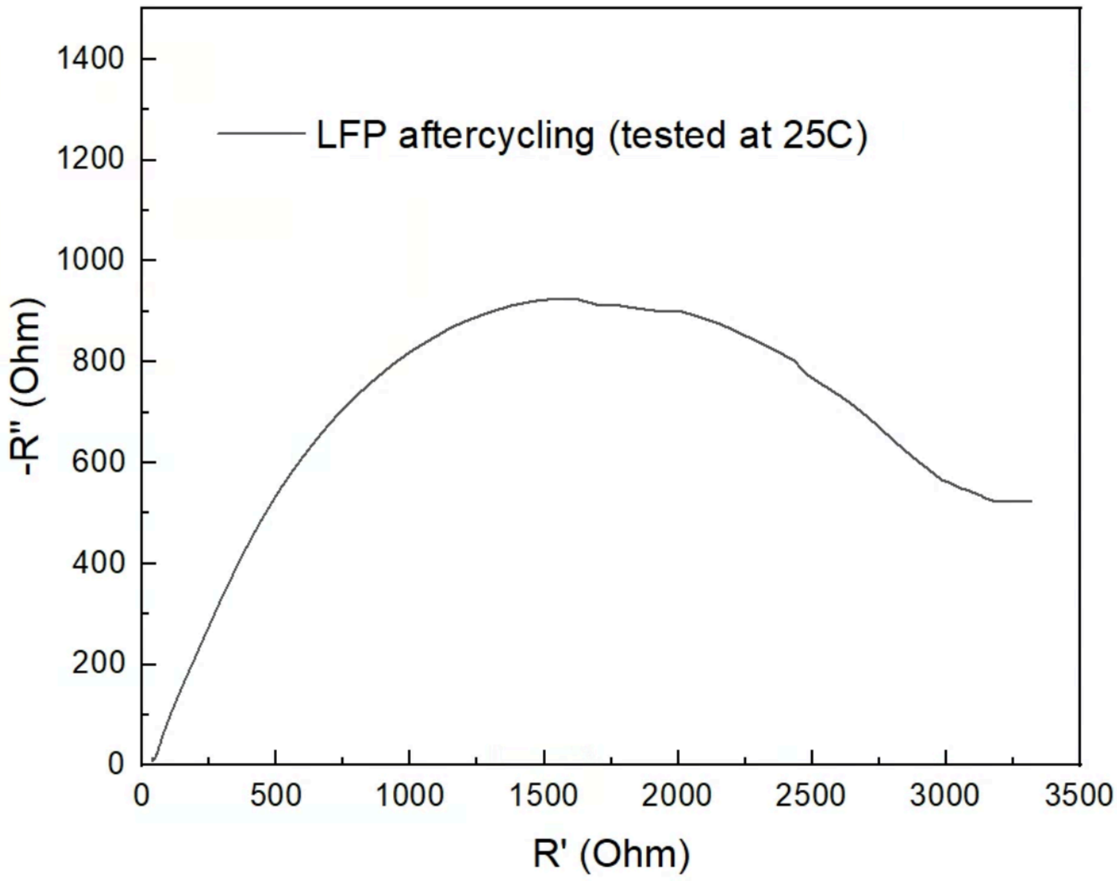

Figure S10. EIS of the LFP||Li cell after cycled at 100◦C for 100 cycles. The EIS measurement was performed at 25◦C after the cell was cooled down.

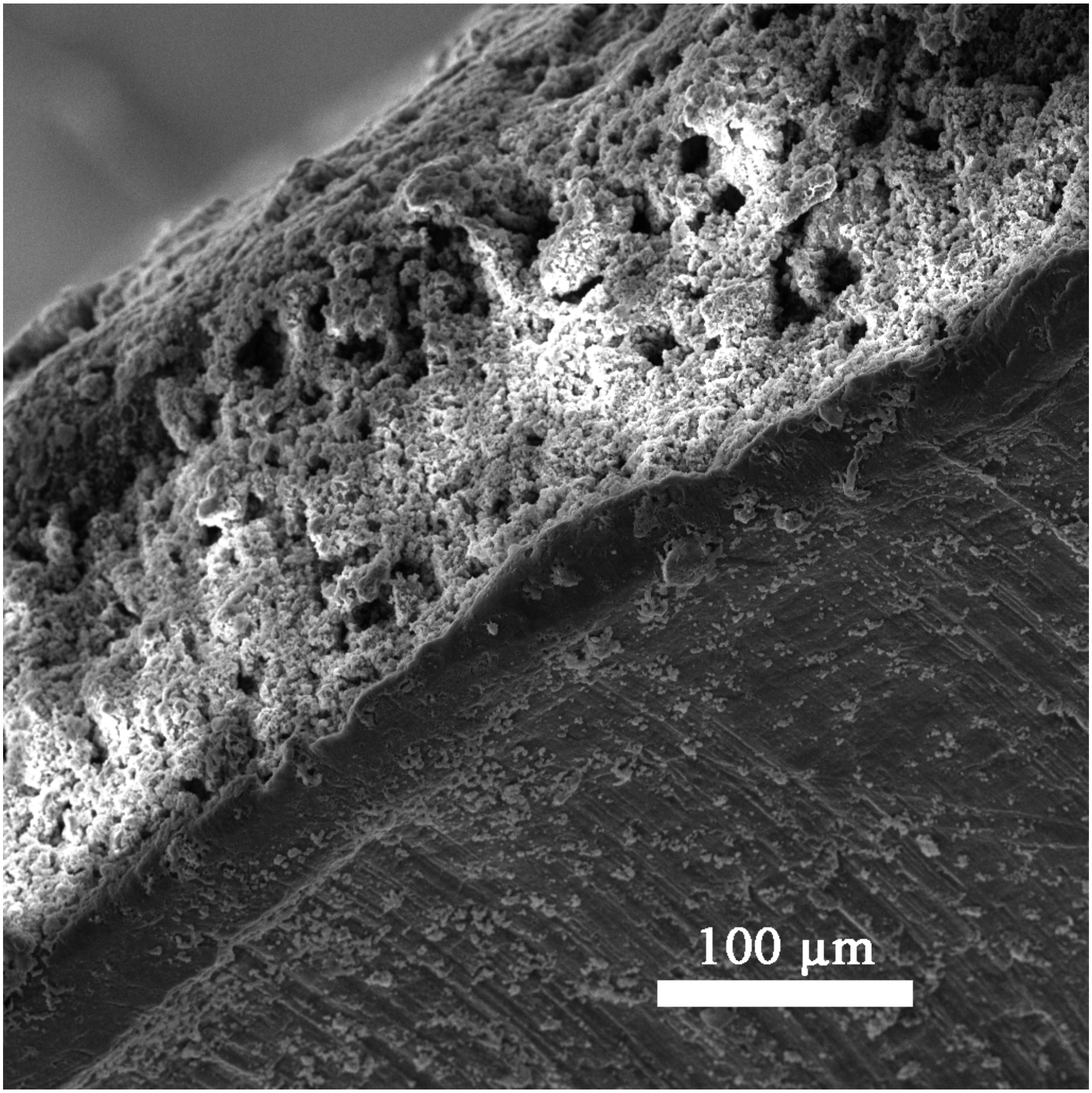

Figure S11. SEM image of the lithium metal off the LFP||Li cell after cycled at 100◦C for 100 cycles.

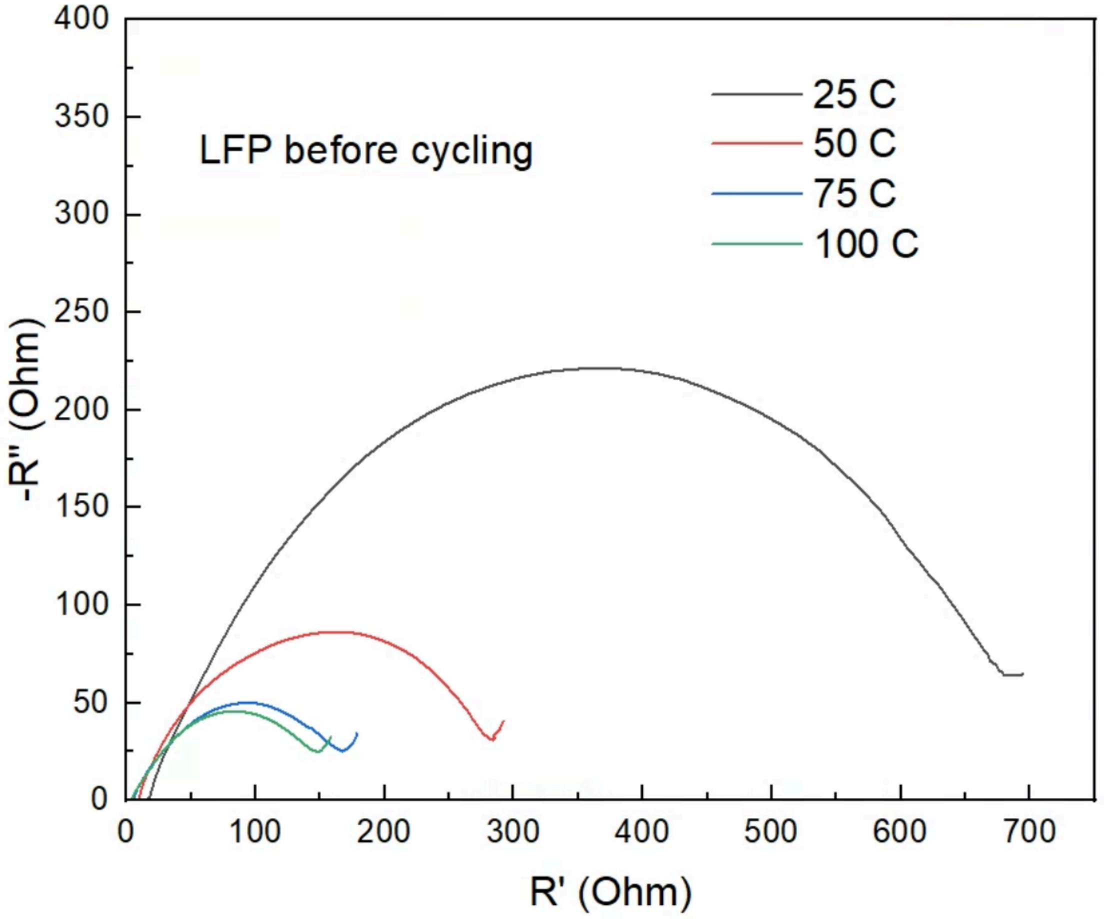

Figure S12. EIS of the LFP||Li cell before cycling. The EIS measurement was performed at different temperatures listed on the plot.

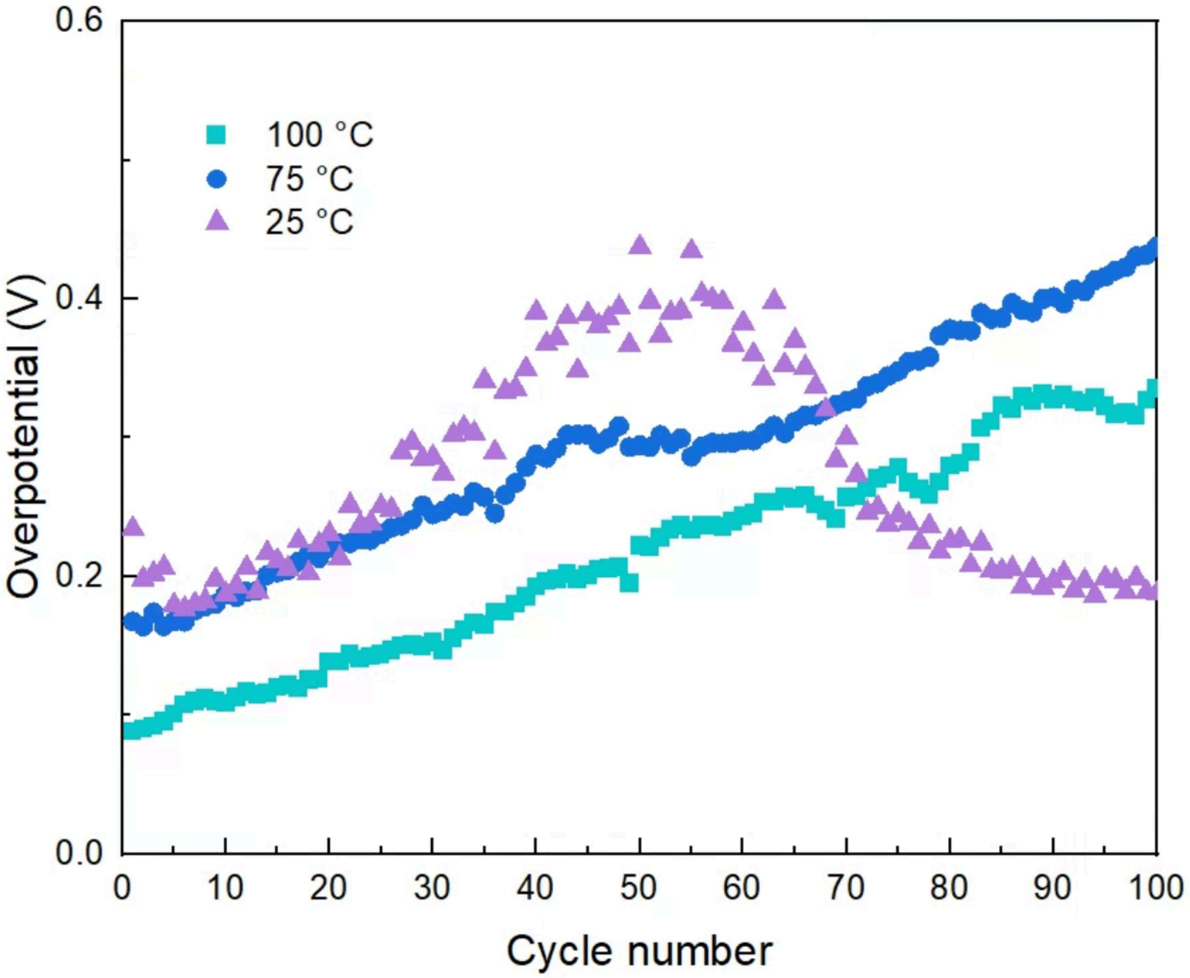

Figure S13. The comparison of the overpotential evolution during cycling of the LFP||Li cells using G2 electrolyte cycled at a 0.2C rate at different temperatures.

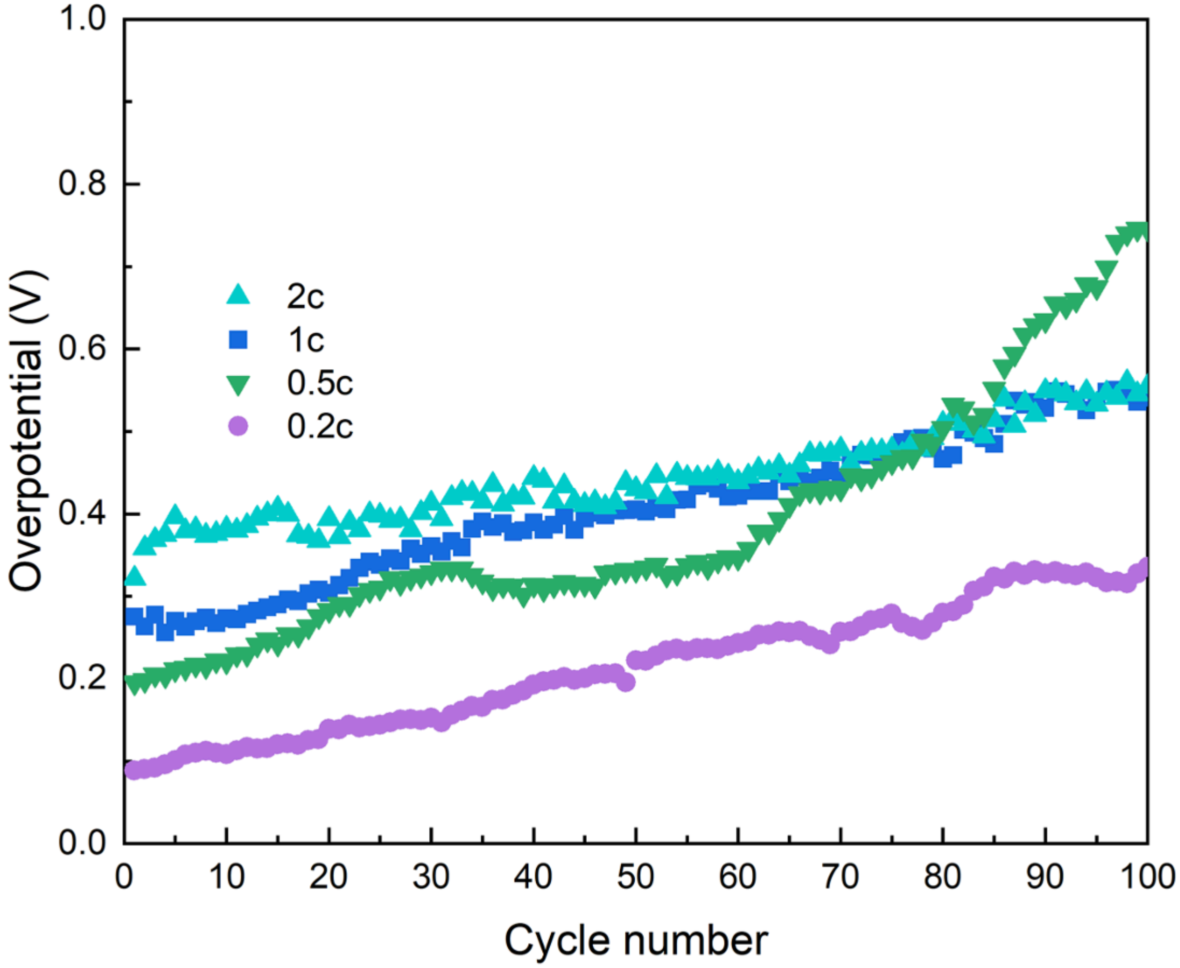

Figure S14. The comparison of the overpotential evolution during cycling of the cells using G2 electrolyte cycled at different C-rates at 100◦C in LFP half cells.

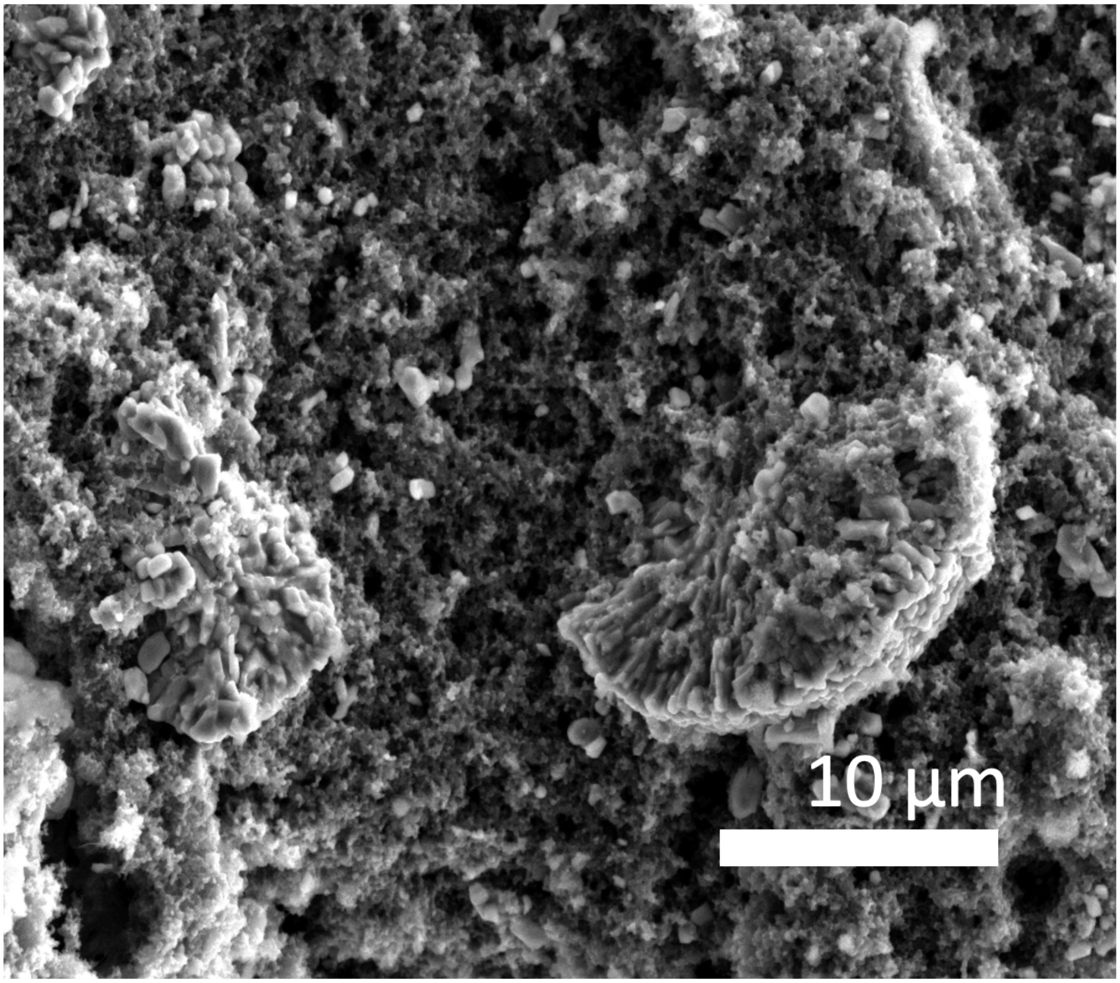

Figure S15. The SEM image of the NCM523 cathode after cycling at 1C at 100 °C.

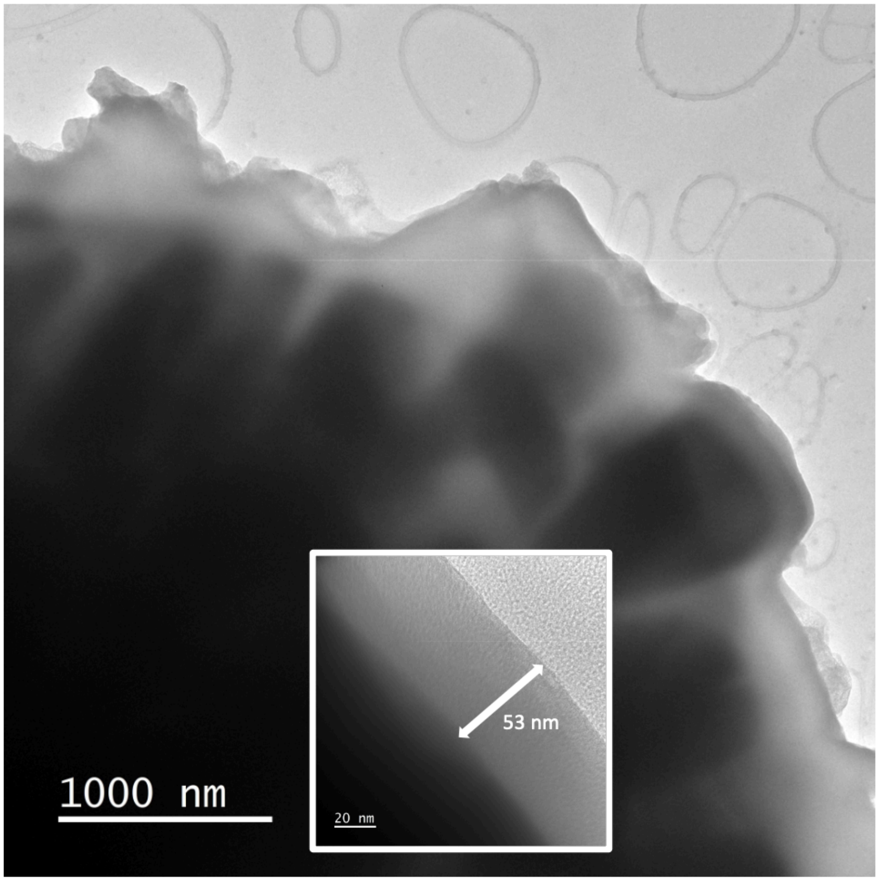

Figure S16. The TEM image of the NCM523 cathode after cycling at 1C at 100 °C.

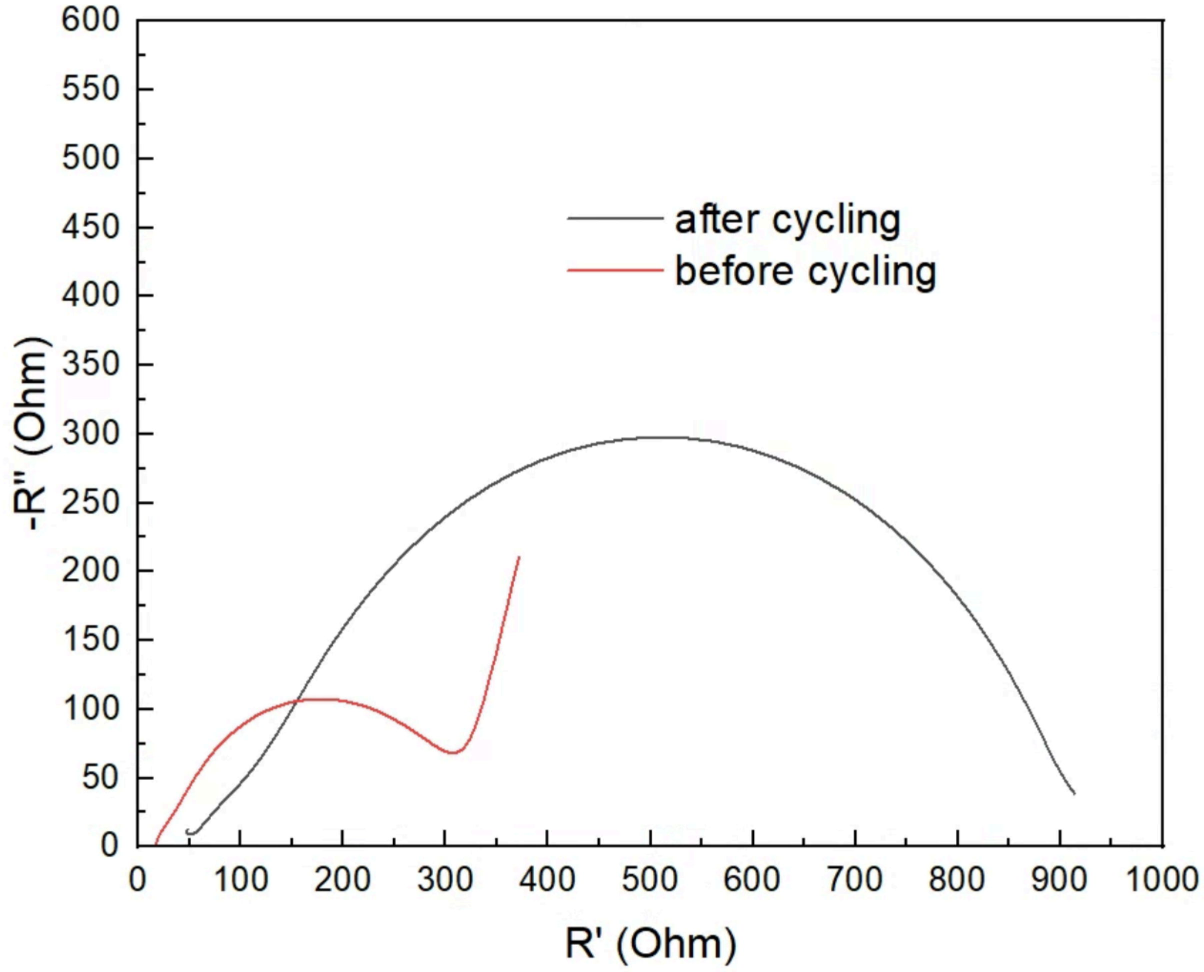

Figure S17. EIS of the NCM523||Li cell before and after cycled at 100°C for 100 cycles. The EIS measurement was performed at 25◦C after the cell was cooled down.

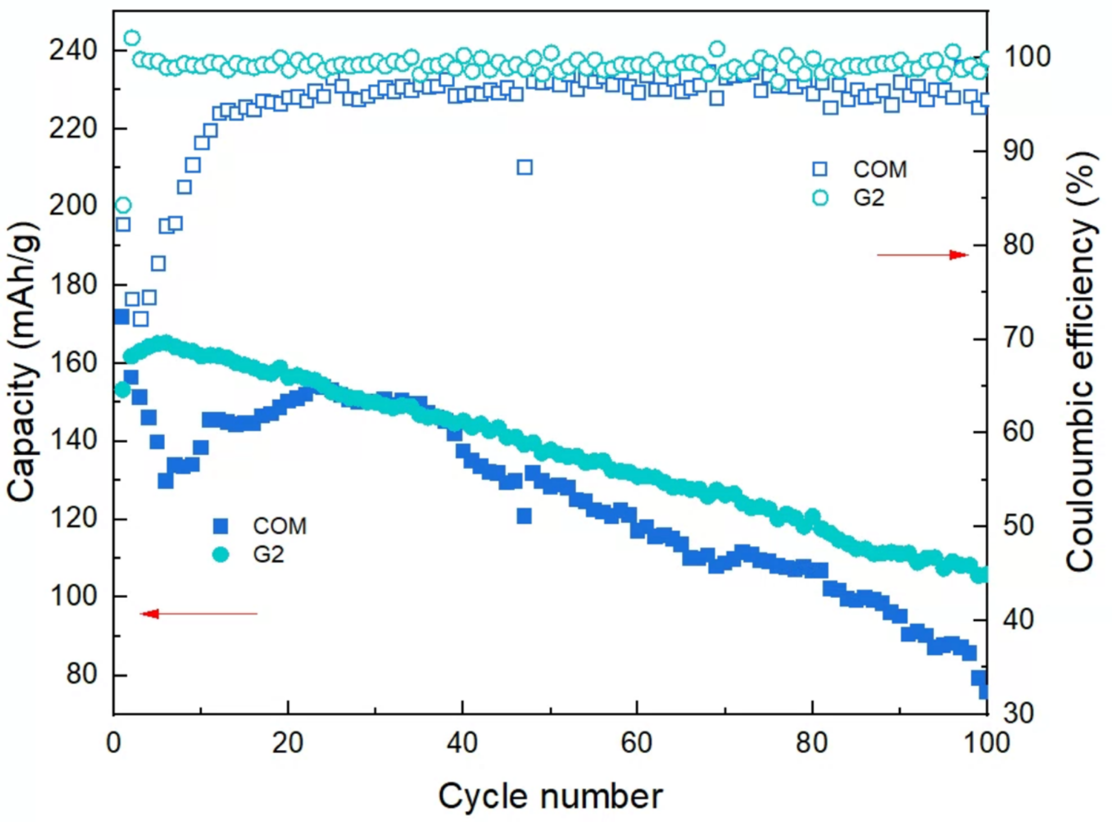

Figure S18. The capacity retention and CE plot of the NCM523||Li cells using G2 and COM electrolyte cycled at a 2C rate at 100 °C.

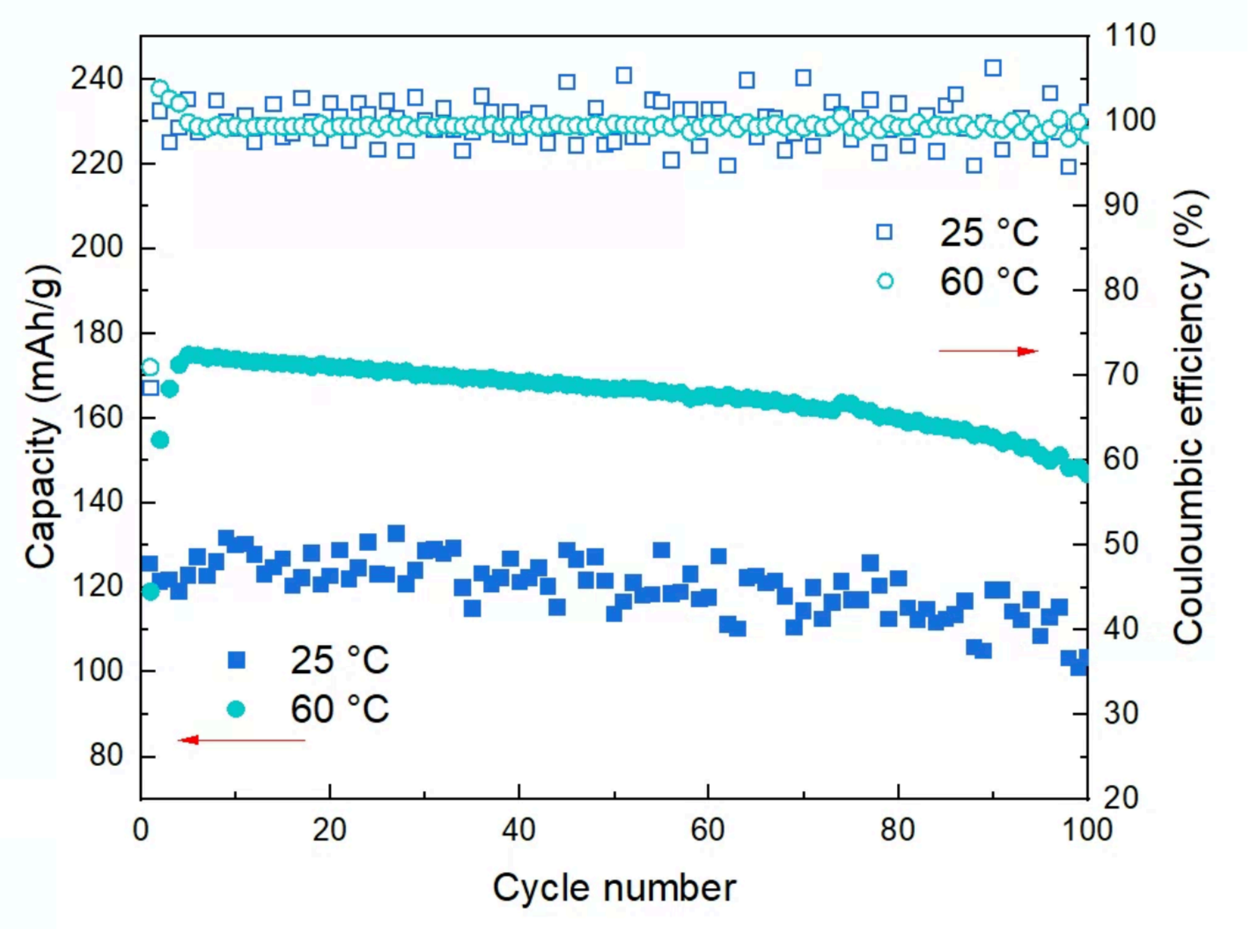

Figure S19. The capacity retention and CE plot of the NCM523||Li cells using G2 electrolyte cycled at a 0.2C rate at 25 °C and 60 °C.

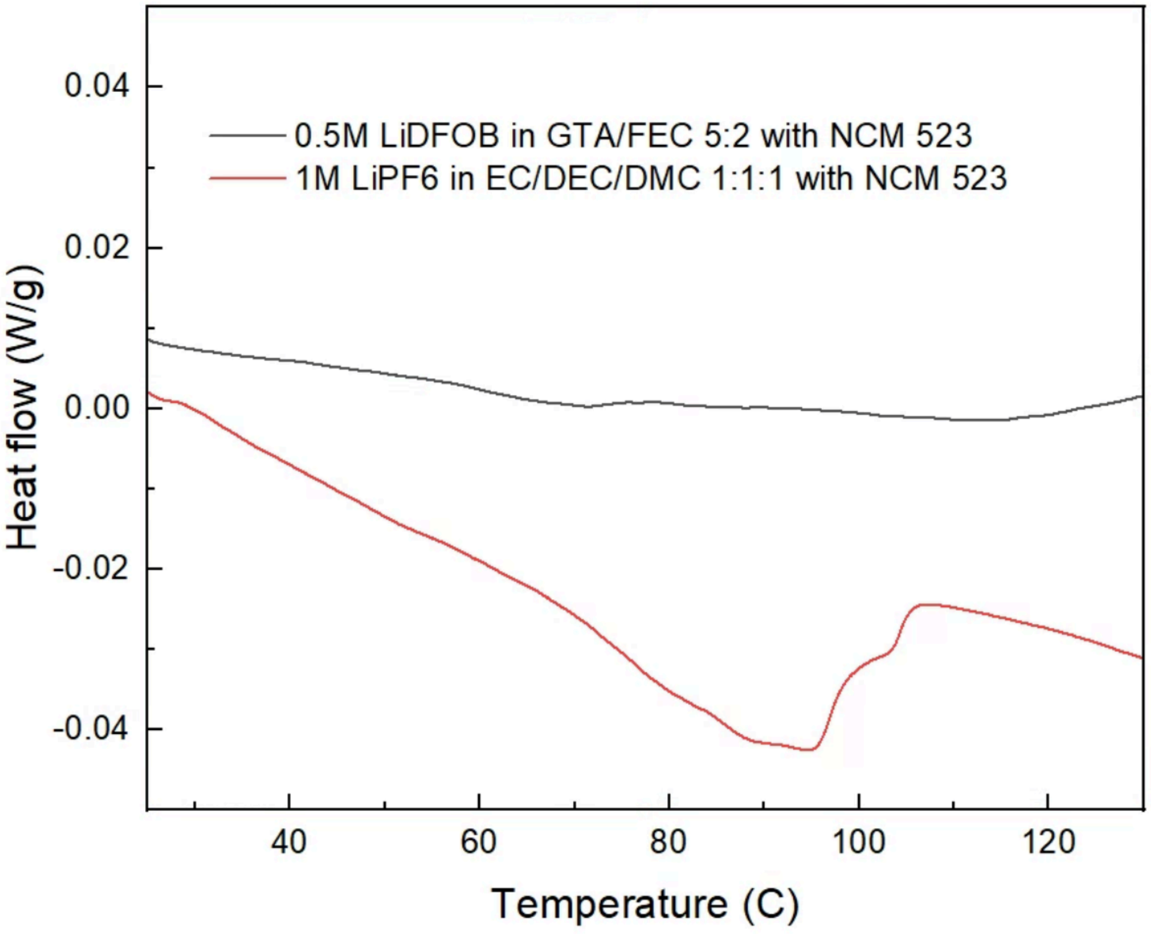

Figure S20. DSC results of the COM and G2 electrolyte with the NCM523 cathode material

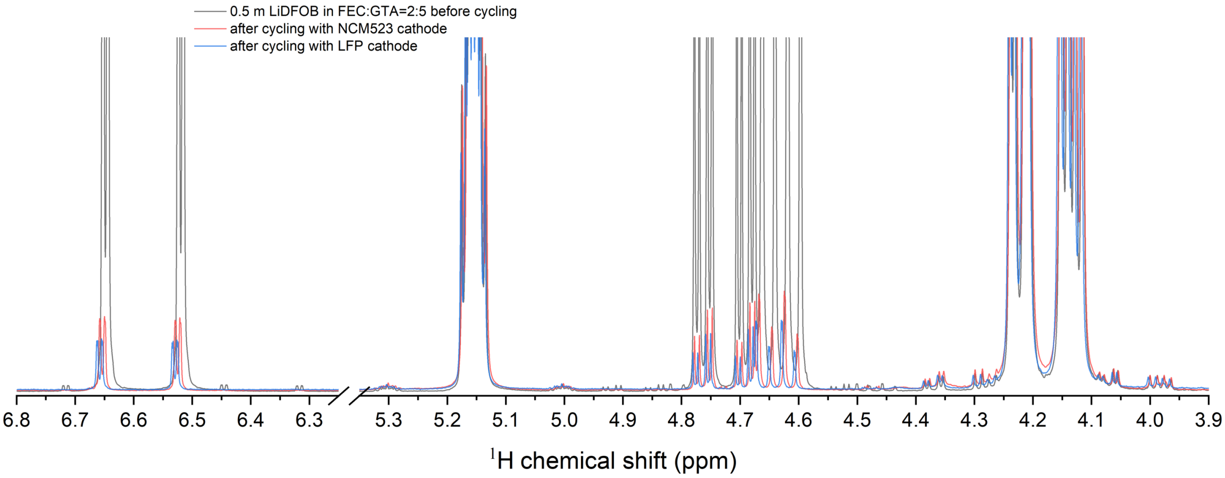

Figure S21. ^{1}H NMR spectrum of the G2 electrolyte before cycling and after cycling at 100 °C with LFP||Li and NCM523||Li cells respectively.

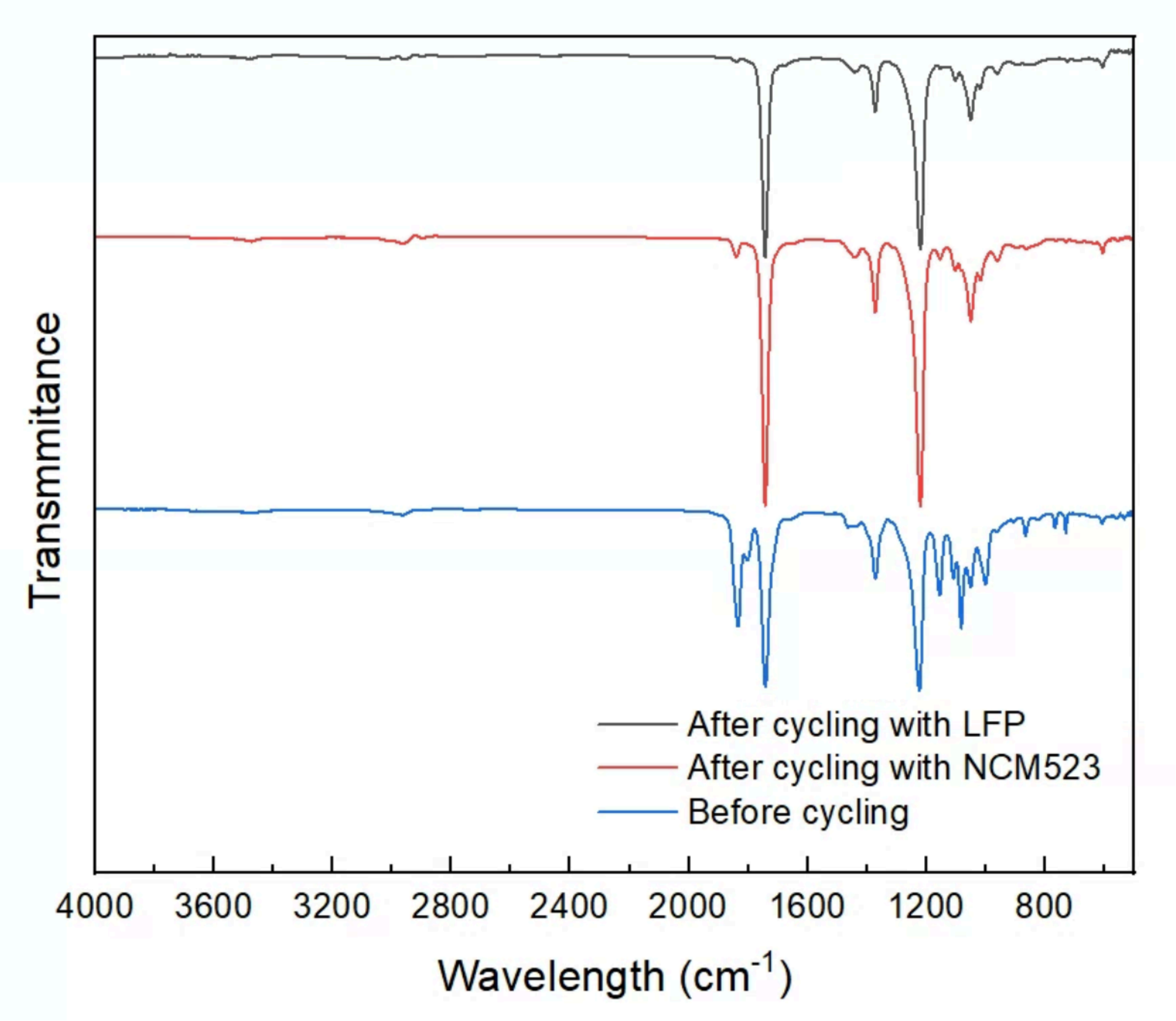

Figure S22. FTIR spectrum of the G2 electrolyte before cycling and after cycling at 100 °C with LFP||Li and NCM523||Li cells respectively

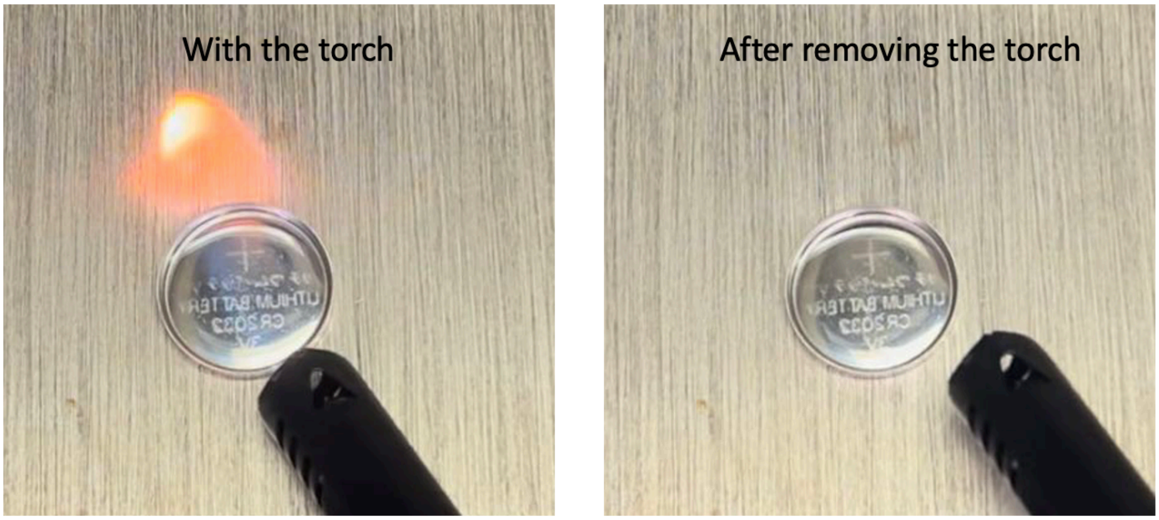

Figure S23. Direct firing test of the G2 electrolyte with the torch.

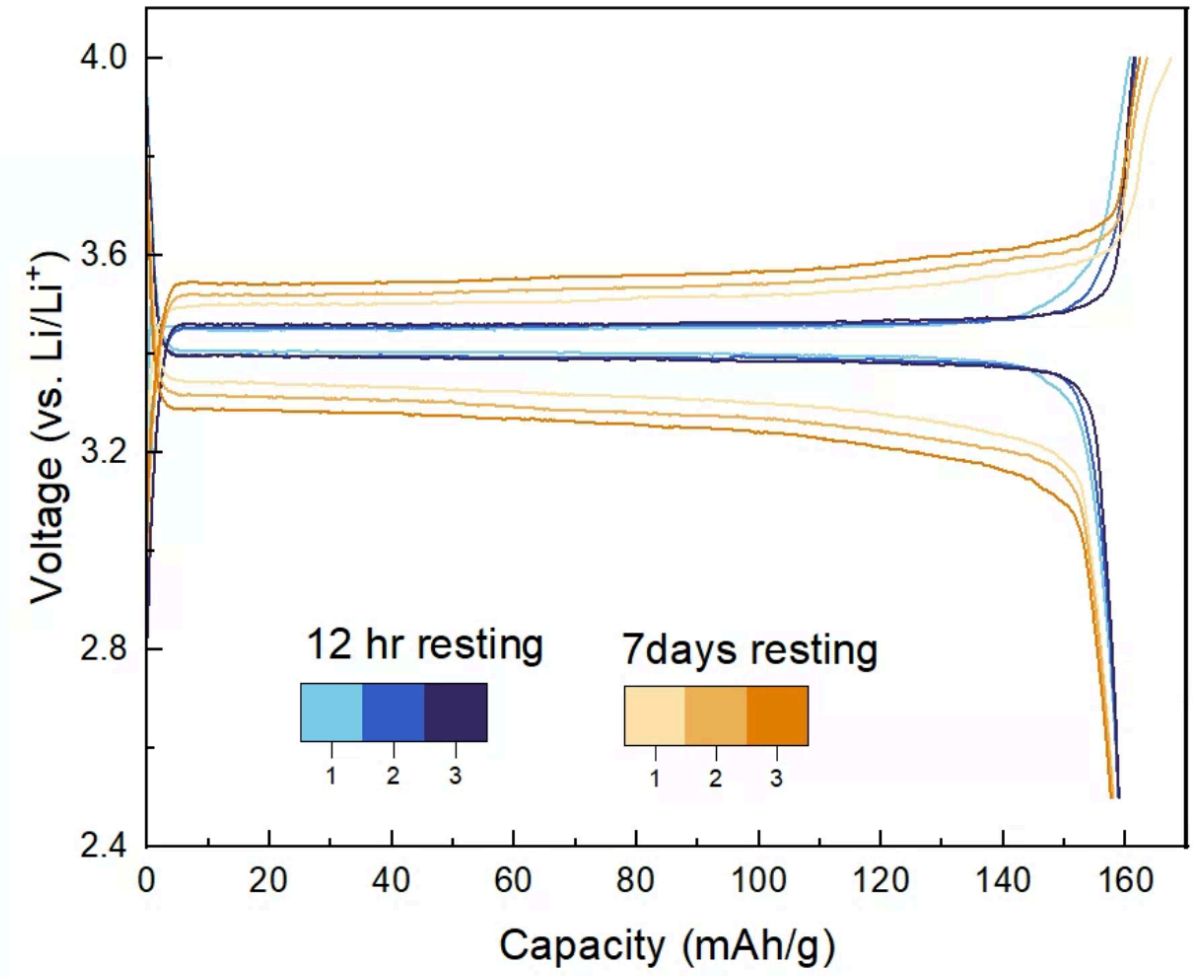

Figure S24. The first three charge-discharge curve of the LFP||Li cell using G2 electrolyte after different resting time at 100C.

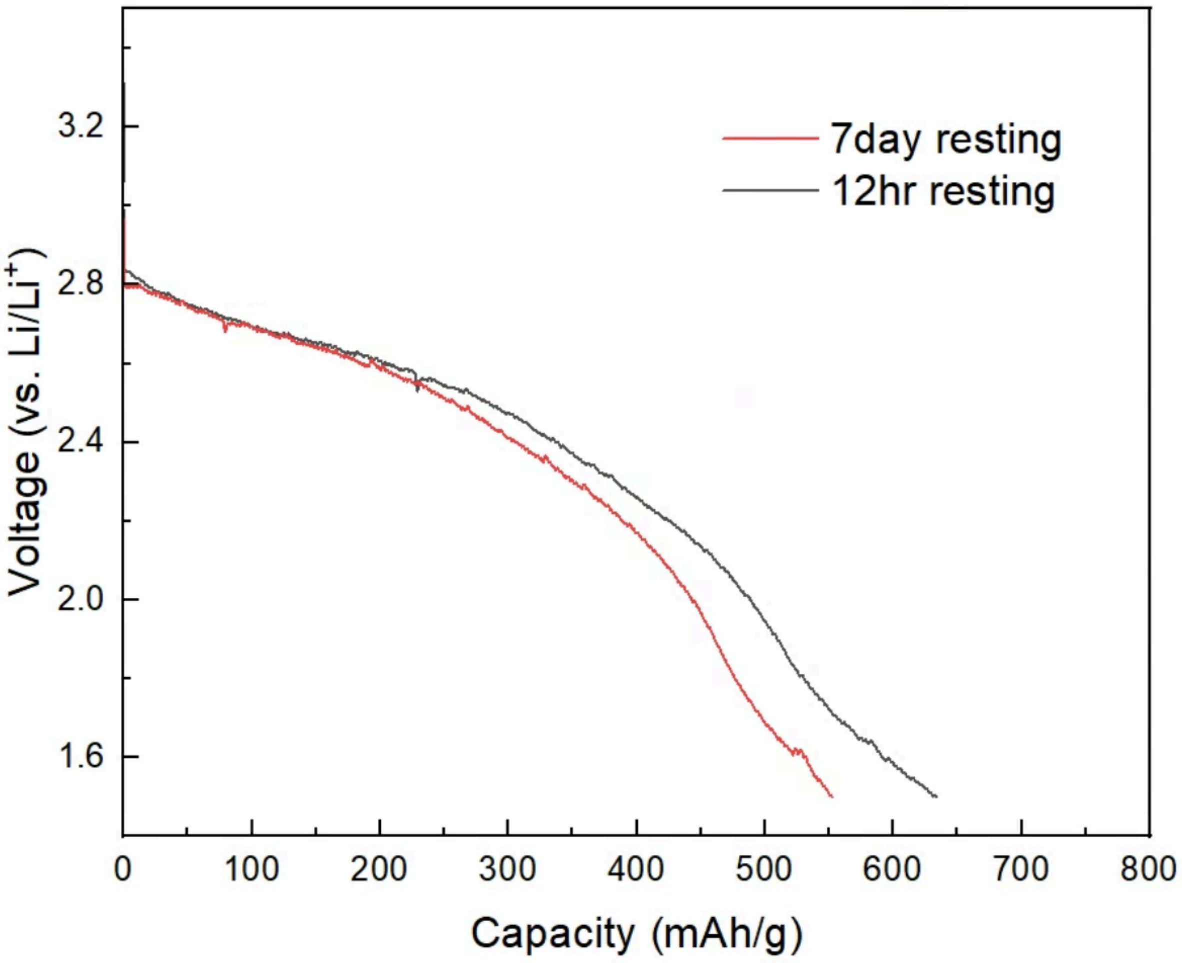

Figure S25. The discharge curve of the Li-CFx||Li cell using COM electrolyte after different resting time at 100C.

	Conductivity (mS/cm)		
Temperature	G1	G2	Commercial
25 °C	1.01	0.61	8.33
30 °C	1.44	0.79	8.94
40 °C	2.28	1.14	10.18
50 °C	3.27	1.34	11.44
60 °C	4.15	1.77	12.79
70 °C	5.35	2.47	14.50
80 °C	6.31	2.95	15.41
90 °C	6.70	3.18	16.74
100 °C	6.85	4.09	17.38

Table S1. The calculated conductivity of the G1, G2, and commercial electrolyte at different temperatures.

Solvent	Salt	Operation temperature (°C)	Cathode active material	Cycle number	Capacity retention rate
FEC:TEGDME = 3:7 (v/v) [1]	2.0 M LiFSI and 0.2 M $LiNO_3$	90	LFP	100 cycles	91.5% @ 1C
TEGDME[2]	LiFSI + LiNO3 1:1 wt%	100	LFP	50 cycles	89% @ 0.2C
Pyr13TFSI : FEC = 0.6:0.2[3]	0.3 M LiTFSI	80	LFP	20 cycles	99% @ 0.2C
DMC[4]	4M LiFSA	100	NCM	100 cycles	66% @ 2C
TEP + 2% FEC[5]	1M LiTFSI	100	NCM	50 cycles	~75% @ 1C
FEC:GTA = 2:5 (v/v) (This work)	0.5M LiDFOB	100	LFP	100 cycles	95.6% @ 0.2C
FEC:GTA = 2:5 (v/v) (This work)	0.5M LiDFOB	100	NCM	100 cycles	65.6% @ 1C

Table S2. Comparison with different reported high-temperature electrolyte systems

$$\sigma = \frac{F_{cali}}{R} \qquad (Equation\ S1)$$

F_{cali} was calibrated with a conductivity standard from HANNA instruments.

$$V_{ov} = \frac{1}{Q_{charge}}\int_0^{Q_{charge}} V_{charge}dq - \frac{1}{Q_{discharge}}\int_0^{Q_{discharge}} V_{discharge}dq \qquad (Equation\ S2)$$

$$CE = \frac{\int_0^{Q_{discharge}} V_{discharge}dq}{\int_0^{Q_{charge}} V_{charge}dq} \qquad (Equation\ S3)$$

(1) Hou, L.; Zhang, X.; Li, B.; Zhang, Q.; Hou, L.; Zhang, X.; Li, B.; Zhang, Q. Cycling a Lithium Metal Anode at 90 °C in a Liquid Electrolyte. *Angew. Chem. Int. Ed.* **2020**, *59* (35), 15109–15113.
(2) Chen, T.; Jin, Z.; Liu, Y.; Zhang, X.; Wu, H.; Li, M.; Feng, W.; Zhang, Q.; Wang, C. Stable High-Temperature Lithium-Metal Batteries Enabled by Strong Multiple Ion–Dipole Interactions. *Angew. Chem. Int. Ed.* **2022**, *61* (35), e202207645.
(3) Plylahan, N.; Kerner, M.; Lim, D. H.; Matic, A.; Johansson, P. Ionic Liquid and Hybrid Ionic Liquid/Organic Electrolytes for High Temperature Lithium-Ion Battery Application. *Electrochim. Acta* **2016**, *216*, 24–34.
(4) Wang, J.; Zheng, Q.; Fang, M.; Ko, S.; Yamada, Y.; Yamada, A. Concentrated Electrolytes Widen the Operating Temperature Range of Lithium-Ion Batteries. *Adv. Sci.* **2021**, *8* (18), 2101646.
(5) Jiang, L.; Liang, C.; Li, H.; Wang, Q.; Sun, J. Safer Triethyl-Phosphate-Based Electrolyte Enables Nonflammable and High-Temperature Endurance for a Lithium Ion Battery. *ACS Appl. Energy Mater.* **2020**, *3* (2), 1719–1729.
